# Single-cell transcriptome analysis and in vitro differentiation of testicular cells reveal novel insights into male sterility of the interspecific hybrid cattle-yak

**DOI:** 10.1186/s12864-023-09251-2

**Published:** 2023-03-27

**Authors:** TserangDonko Mipam, Xuemei Chen, Wangsheng Zhao, Peng Zhang, Zhixin Chai, Binglin Yue, Hui Luo, Jikun Wang, Haibo Wang, Zhijuan Wu, Jiabo Wang, Mingxiu Wang, Hui Wang, Ming Zhang, Hongying Wang, Kemin Jing, Jincheng Zhong, Xin Cai

**Affiliations:** 1 Key Laboratory of Qinghai-Tibetan Plateau Animal Genetic Resource Reservation and Utilization, Sichuan Province and Ministry of Education, Southwest Minzu University, Chengdu, 610041 Sichuan China; 2 School of Life Science and Engineering, Southwest University of Science and Technology, Mianyang, 621010 Sichuan China; 3 College of Chemistry & Environment, Southwest Minzu University, Chengdu, 610041 Sichuan China

**Keywords:** Cattle-yak, Male sterility, Single-cell transcriptome, Differentiation, Testicular cells

## Abstract

**Background:**

Interspecific hybridization plays vital roles in enriching animal diversity, while male hybrid sterility (MHS) of the offspring commonly suffered from spermatogenic arrest constitutes the postzygotic reproductive isolation. Cattle-yak, the hybrid offspring of cattle (*Bos taurus*) and yak (*Bos grunniens*) can serve as an ideal MHS animal model. Although meiotic arrest was found to contribute to MHS of cattle-yak, yet the cellular characteristics and developmental potentials of male germline cell in pubertal cattle-yak remain to be systematically investigated.

**Results:**

Single-cell RNA-seq analysis of germline and niche cell types in pubertal testis of cattle-yak and yak indicated that dynamic gene expression of developmental germ cells was terminated at late primary spermatocyte (meiotic arrest) and abnormal components of niche cell in pubertal cattle-yak. Further in vitro proliferation and differentially expressed gene (DEG) analysis of specific type of cells revealed that undifferentiated spermatogonia of cattle-yak exhibited defects in viability and proliferation/differentiation potentials.

**Conclusion:**

Comparative scRNA-seq and in vitro proliferation analysis of testicular cells indicated that not only meiotic arrest contributed to MHS of cattle-yak. Spermatogenic arrest of cattle-yak may originate from the differentiation stage of undifferentiated spermatogonia and niche cells of cattle-yak may provide an adverse microenvironment for spermatogenesis.

**Supplementary Information:**

The online version contains supplementary material available at 10.1186/s12864-023-09251-2.

## Background

Interspecific hybridization plays vital roles in enriching biological diversity and adaptation, while the sterility of male hybrid offspring often restricts transmitting of the excellent genotypes to subsequent generations. MHS was generally attributed to incompatibility of genomic loci from the parental lineages [1]. According to Haldane’s rule, MHS resulted when recessive X-linked alleles were expressed in hemizygous (XY or ZW) hybrids but masked in their heterozygous (XX or ZZ) hybrid siblings [2]. MHS factors were first localized to chromosomal regions and the X chromosome exhibited a disproportionately large effect on the fertility of hybrid males [3]. Several decades later, dominance and epistasis theory were developed and assumed that there were more than one case of a fully dominant MHS factor and epistasis modified the dominance of MHS factors [4, 5]. The last two decades of work on molecular basis of MHS identified genes showed patterns of substitution consistent with recurrent positive selection and evolutionary conflicts [6]. Furthermore, a population genetic theory was developed based on Dobzhansky-Muller model that MHS could also result from the transposition of gene function from one genomic address to another in different lineages [7].

Among thousands of mammalian species, cattle-yak, the hybrid offspring of cattle (*Bos taurus*) and yak (*Bos grunniens*), is known to be an ideal animal model to study the mechanism of MHS. Currently, MHS of cattle-yak was generally regarded to be caused by meiotic arrest at about the pachytene stage due to abnormal gene expression and epigenetic regulations. The downregulation of meiotic related genes *SYCP3*, *DDX4*, *DMC1* and *DMRT7* was associated with spermatogenic arrest and MHS of cattle-yak [8–10]. Abnormal expressions and localizations of H3K4me3, H3K9me1, H3K9me3 and H4K20me3 in meiotic chromosomes of cattle-yak spermatocytes suggested their potential roles in spermatogenic failure and hybrid male sterility [11]. Promoter hypermethylation of PIWI/piRNA pathway genes was found to be associated with diminished pachytene piRNA production in cattle-yak [12]. Cattle-yak hybrids showed higher 5MC expression levels and different AcK9 expression levels in all cell types compared to the same-aged yak, which was proposed to be the factors for disruption of normal germ cell development in cattle-yak [13]. In our previous work, comparative transcriptomic and proteomic profiles in whole-mount testis revealed abnormal gene and protein expressions in both spermatogenic and somatic cells of cattleyak, and aberrant miRNA and genomic DNA methylation were observed in cattle-yak spermatogenic cells, which indicated that spermatogenic arrest of cattle-yak might occur as early as the differentiation stage of spermatogonia stem cells and be aggravated during meiosis [14–17]. Sato et al. (2020) also identified the decreased expression of AR and increased expression of 3βHSD in the Leydig cells of cattle-yak hybrid testes [18]. Notably, disruption of spermatogenic & Leydig cell proliferation and apoptosis balance was revealed in the testes of cattle-yaks [19]. However, the cellular characteristics and developmental potentials of male germline and niche cell in pubertal cattle-yak remain to be systematically investigated.

In recent years, the single-cell RNA sequencing (scRNA-seq) technique was used to delineate the heterogeneity and developmental landscapes of adult testis and germlines in several mammalian species including sheep and goat [20–23]. In pubertal mammals, “testicular puberty” is triggered with meiotic entry of differentiated spermatogonia B to form the primary spermatocytes, followed by their subsequent maturation into spermatozoa [24]. The initiation of steady-state spermatogenesis was observed in pubertal yak testis aged 24 months, resulting in almost all types of germ cells [25]. Here, we use scRNA-seq to systematically examine germline and niche cell types and dynamic gene expression patterns in pubertal testis of cattle-yak with the yak as control. In addition, we compared the proliferation potentials of undifferentiated spermatogonia (UDSPG) cells between cattle-yak and yak in vitro.

## Results

### Single-cell transcriptome profiling and identification of pubertal testicular cells from cattle-yak and yak

**Fig. 1:**
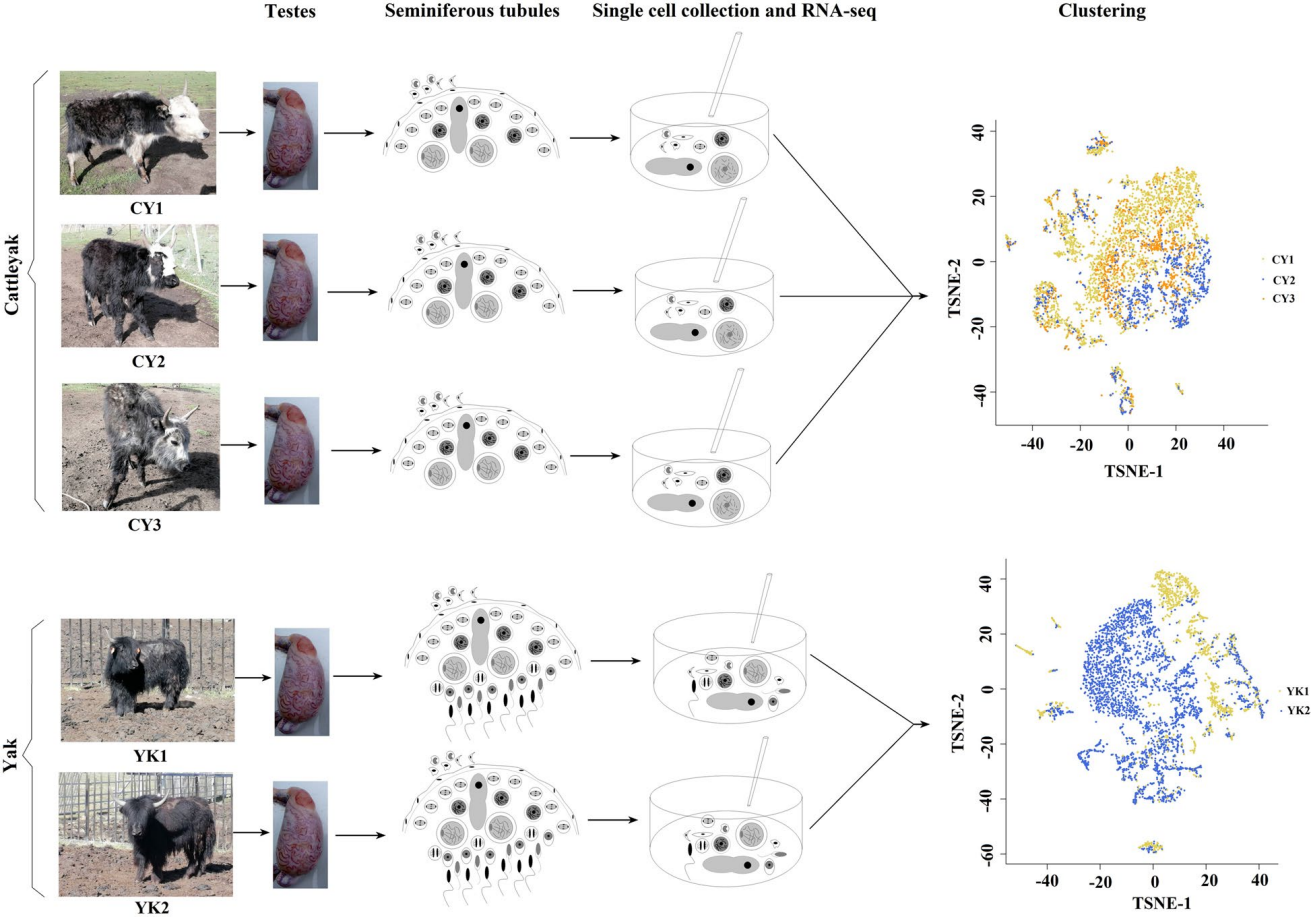
Single-cell transcriptome profiling of pubertal testicular cells from cattle-yak and yak. Schematic illustration of experimental workflow for single-cell transcriptome profiling of testicular cells from cattle-yak and yak. Three cattle-yaks and two yaks were sampled. The testis from each individual was obtained by veterinary surgical operation and used for single-cell isolation and transcriptome analysis. CY and YK denote cattle-yak and yak, respectively

Single-cell isolation was performed following the standard protocol of enzyme digestion and physical filtering from the testes of pubertal cattle-yak and yak with three and two biological replications, respectively [16, 26] (Fig. 1). In total, 9988 testicular cells from five individuals passed standard quality control and kept for the subsequent scRNA-seq analysis. The mapping rate ranged from 92.5% to 93.5% and averaged 92.9%; the mean reads per cell ranged from 117,866 to 352,053 and averaged 246,498; the gene numbers per cell ranged from 15,936 to 18,628 and averaged 17,331 (Table S1).

**Fig. 2:**
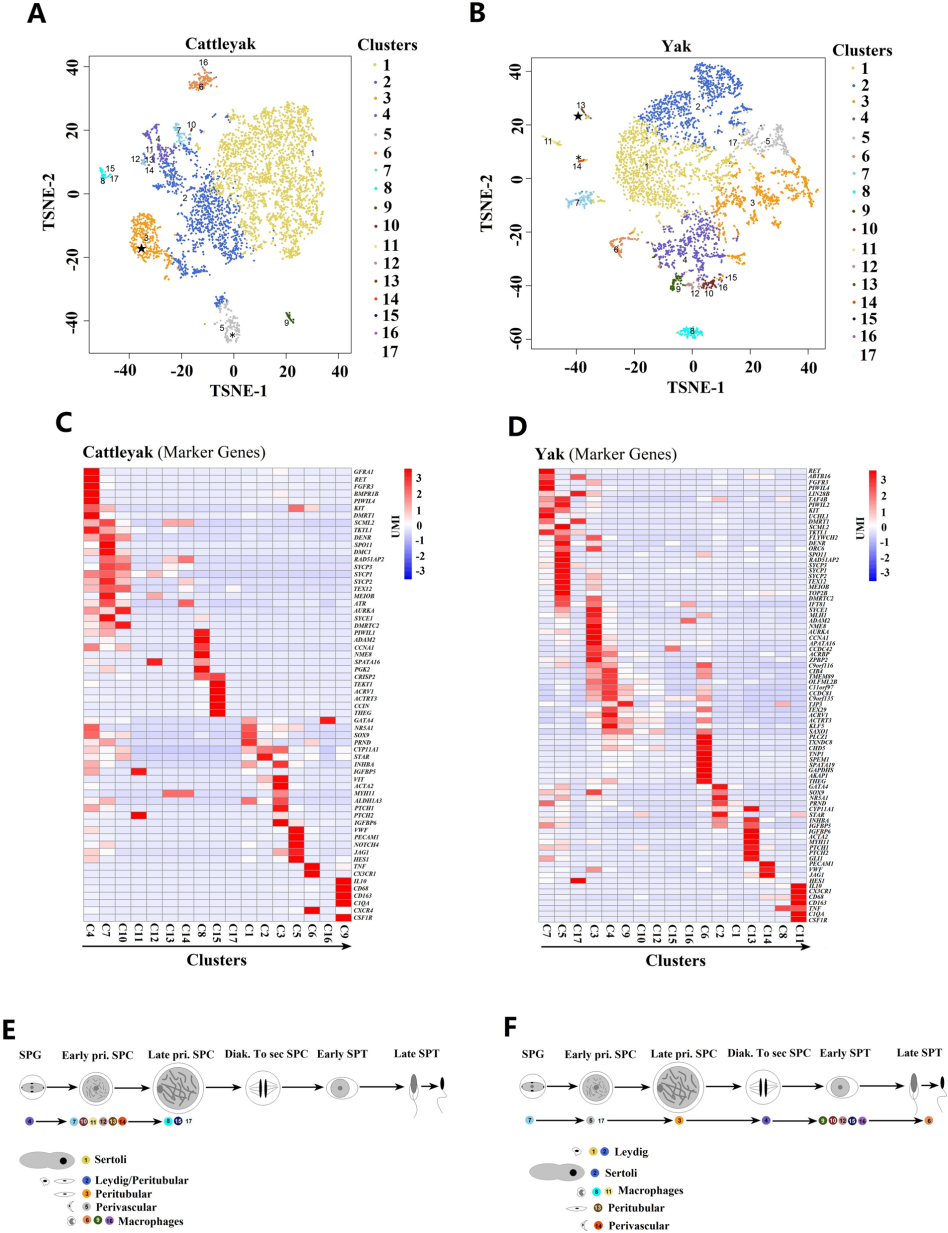
Coordinated gene expression and testicular cell-type identification in pubertal cattle-yak and yak. **A**, **B** tSNE plots of 10 × Genomics profiling of testicular cells from cattle-yak and yak, respectively. **C**, **D** Heatmap for the cell-type specific gene expressions between each cell cluster in cattle-yak and yak testis, respectively. **E**, **F** Assignment of pubertal testicular cell types according to the identified cell clusters by cell specific signature genes in cattle-yak and yak, respectively. The color gradient from red to blue shows high to low expression levels. The pentagram and asterisk indicate peritubular and perivascular cells, respectively

Unsupervised clustering by t-distributed stochastic neighbor embedding (tSNE) analysis revealed that cattle-yak and yak testicular cells presented different distribution patterns of cell populations (Fig. 1). We identified 17 clusters of testicular cells in both cattle-yak (Fig. 2A; Table S2) and yak (Fig. 2B; Table S3). Cell clusters from the two bovid species were assigned on the basis of cell-type specific gene expression during spermatogenesis (Fig. 2C and D). Among the 17 cell clusters, niche cells took up over 95% of all the testicular cells in cattle-yak (clusters 1, 2, 3, 5, 6, 9, 16) (Fig. 2A and C) and 50% in yak (clusters 1, 2, 8, 11, 13, 14) (Fig. 2B and D). Meanwhile, fewer germ cells (Clusters 4, 7, 10–14, 8, 15 and 17) (Fig. 2A and C) were identified in cattle-yak than those in yak (Clusters 7, 5, 17, 3, 4, 9, 10, 12, 15, 16 and 6) (Fig. 2B and D). To validate the divergences in cell number and proportions between cattle-yak and yak, testicular tissues from each bovid species were digested with mixed enzymes and adhered to the dishes for 24 h, then the supernatant was collected and the germ cells were identified by DDX4 immunofluorescence staining. The density of cattle-yak testicular cells was lower than that of yak (Fig. S1A and S1B), and the size of these mixed cells of cattle-yak was also smaller than that of yak (Fig. S1C and S1D). Cattle-yak also presented lower proportion of DDX4^+^ germ cells compared to yak (Figure S1E and S1F).

### Coordinated gene expression and testicular cell-type identification in cattle-yak and yak

All the germ cells identified in cattle-yak and yak exhibited coordinated expression of specific signature genes. Cluster 4 corresponded to spermatogonia (SPG) in cattle-yak and presented the specific expression of known SSC (*GFRA1*, *RET*, *FGFR3* and *BMPR1B*) and differentiating SPG marker genes (*KIT* and *DMRT1*) (Fig. 2C) [20, 21, 27]. Clusters 7, 10–14 were assigned as early primary spermatocytes (pri. SPC) in cattle-yak for the expression of markers in meiotic recombination (*SPO11*, *DMC1* and *MEIOB*), synaptonemal complex (SC) components (*SYCP3*, *SYCP1*, *SYCP2*, *TEX12* and *SYCE1*), SC disassembly (*AURKA*) and other meiosis involved genes (*ATR*, *DMRTC2*) (Fig. 2C) [28–32]. Noticeably, clusters 8, 15 and 17 corresponding to late pri. SPC terminated the development process of germ cells in cattle-yak, in which we detected the expression of markers identified from pachytene to the early stage of spermatids (*PIWIL1*, *ADAM2*, *CCNA1*, *NME8*, *SPATA16* and *PGK2)* [21, 33, 34]*.* Accordingly, these known marker genes were also specifically expressed in SPG (cluster 7), pri. SPC (clusters 5 and 17) and late pri. SPC (cluster 3) of yak (Fig. 2D). To be entirely different from cattle-yak, marker genes were coordinatedly expressed in developmentally consecutive germ cells of diakinesis to secondary spermatocytes (Diak. To sec. SPC), Early spermatids (SPT) and Late SPT in yak. The genes involved in late stage of meiosis (*C9orf116*), haploid differentiation (*CIB4*) and sperm centrosome conformation (*CCDC81*) were highly expressed in Diak. To sec. SPC [12, 35, 36]. Furthermore, we observed the upregulation of genes associated with early spermatid development (*TJP3*, *TEX29* and *SAXO1*), acrosomal vesicle components (*ACRV1*), fertilization (*PLCZ1*) and spermatid chromatin remodeling (*TXNDC8* and *CHD5*) [37–39] in early SPT. We also found late SPT exhibited the prominent expressions of genes involved in sperm nuclear condensation (*TNP1*), proper cytoplasm removal (*SPEM1*), organization and function of the mitochondria (*SPATA19*), energy production and sperm motility (*GAPDHS*) and spermatid-sertoli cell interaction (*THEG*) [40–43] (Fig. 2D).

On the other hand, all types of niche cells (Sertoli, Leydig, peritubular, perivascular and macrophage cells) were identified in both cattle-yak and yak. Cluster 1 (Fig. 2C) and cluster 2 (Fig. 2D) corresponded to Sertoli cells of cattle-yak and yak, respectively, presented the higher expression of *SOX9*, *NR5A1* and *PRND* [21, 32, 44]. Cluster 2 (Fig. 2C) and cluster 1 (Fig. 2D) were identified as Leydig cells of cattle-yak and yak, respectively, exhibiting the higher expressions of *CYP11A1* and *STAR* [45, 46]. The markers of peritubular myoid cells (*ACTA2* and *MYH11*) were specifically expressed in Cluster 3 for cattle-yak and cluster 13 for yak [32, 47]. *PECAM1* and *VWF* marking perivascular cells were upregulated in Cluster 5 for cattle-yak and cluster 14 for yak [32]. Cluster 6, 9, 16 for cattle-yak and Cluster 8, 11 for yak were observed to be macrophages with a higher expression of *IL10*, *CX3CR1*, *CD68*, *CD163* and *CSF1R* [21, 32, 48].

To validate the continuum germ cells and niche cells captured in single-cell transcriptome data of pubertal testicular cells from cattle-yak and yak, we performed RT-qPCR to conform the specific signature genes coordinatedly expressed in developmentally consecutive germ cells and niche cells. Among the 46 germ cell specific genes, only one SSC signature (*PIWIL4*), five SPG signatures (*DMRT1*, *SCML2*, *TKTL1*, *FLYWCH2* and *DENR*), one for early pri. SPC (*RAD51AP2*) and one for early pri. SPT (*TJP3*) was un-differentially expressed in cattle-yak and yak, while the other 38 genes were downregulated in cattle-yak (Fig. S2A). Noticeably, the downregulated genes in cattle-yak comprising the signature genes for all types of germ cells, especially, the SSC signatures (*LIN28B*, *FGFR3* and *PLZF*) were moderately downregulated in cattle-yak and a series of SPT specific genes were only expressed in yak (Fig. S2A). By contrast, niche cell specific genes exhibited non coordinated expressions between cattle-yak and yak (Fig. S2B). Leydig cell specific genes (*CYP11A1* and *STAR*) presented higher expressions in cattle-yak, while macrophage cell specific genes (*CD68*, *IL10* and *TNF*) were downregulated in cattle-yak (Fig. S2B).

### Dynamic spermatogenic gene expression patterns interrupted in pubertal cattle-yak during germ cell development

**Fig. 3:**
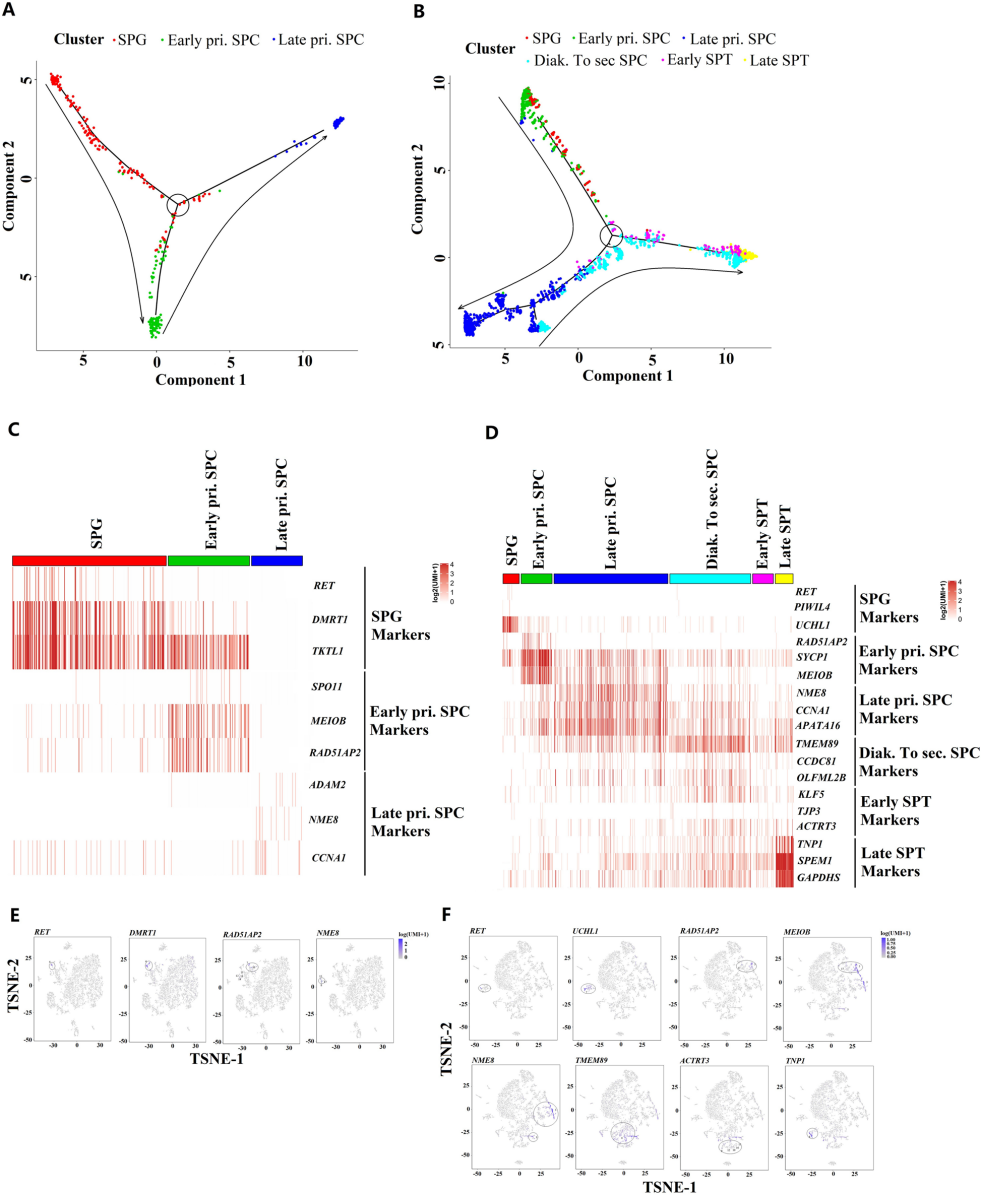
Dynamic spermatogenic gene expression patterns and cell-type specific gene markers in pubertal germ cells of cattle-yak and yak. **A**, **B** Developmental pseudotime of pubertal cattle-yak and yak germ cells, respectively. Cell types were identified from the clusters in corresponding tSNE plots and could be distinguished with different colors. Arrows show the developmental order of the germ cells. **C**, **D** Dynamic expression of selected germ cell signature genes during pubertal cattle-yak and yak spermatogenesis, respectively. The cell types were marked with different color and the cell numbers were showed with different length of bars. The red gradients from dark to light indicate high to low expression level. **E**, **F** Expression patterns of selected germ cell marker genes on tSNE plots of cattle-yak and yak, respectively. The blue gradients from dark to light indicate high to low expression levels

On the basis of dynamic expressions of the marker genes, all types of germ cells were identified in yak, while the dynamic gene expressions during germ cell development were interrupted in cattle-yak at late pri. SPC stage (Fig. 2E and F). Monocle was used to determine the developmental order of germ cells from both cattle-yak and yak. The unsupervised pseudotime analyses yielded the developmental trajectory of cattle-yak and yak spermatogenesis, with both exhibiting major branching from the node of meiosis (Fig. 3A and B). In cattle-yak, the spermatogenesis was apparently arrested at pre-meiotic development stage, for the number of late pri. SPC were sharply reduced and there were no furtherly developed germ cells examined (Fig. 3A). Comparatively, pseudotime exhibited the successive developmental trajectory of germ cells from SPG to Late SPT, with the branching node denoting meiotic entry (Fig. 3B). Furthermore, each germ cell types could be discriminated by several marker genes both in cattle-yak and yak. Only a few of germ cells identified in cattle-yak, dynamic expressions of *RET*, *DMRT1*, *RAD51AP2* and *NME8* in different cell clusters distinguished SPG, early pri. SPC and late pri. SPC, respectively (Fig. 3C and E; Fig. S3A). By contrast, a large numbers of germ cells were identified in yak and dynamic expressions of marker genes in cell clusters could distinguish nearly all types of spermatogenic cells, including *RET* in SPG, *RAD51AP2* in early pri. SPC, *NME8* in late pri. SPC, *TMEM89* in Diak. To sec. SPC, *ACTRT3* in early SPT and *TNP1* in late SPT (Fig. 3D and 3F; Fig. S3B).

**Fig. 4:**
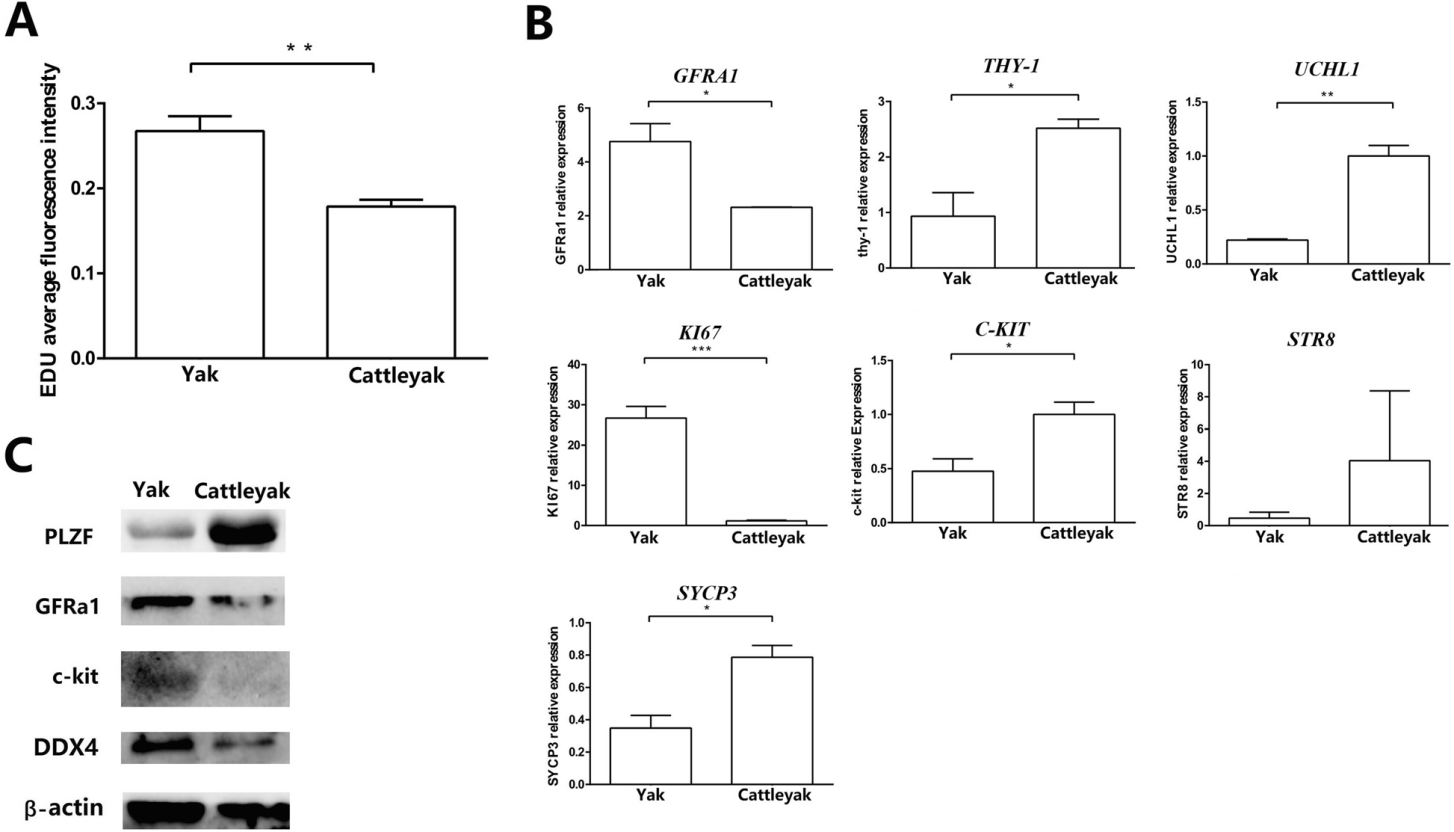
Detection of the proliferation of spermatogenic cells cultured in vitro. **A** The average EDU fluorescence intensity of yak and cattle-yak spermatogenic cells, respectively. **B** RT-qPCR detection of the germ cell mark genes, *GFRA1, THY1, UCHL1, KI67, c-kit, STR8* and *SYCP3* expression in cattle-yak and yak spermatogenic cells cultured in vitro, respectively. **C** Western Blotting detection of the germ cell mark proteins, PLZF, GFRA1, c-kit and DDX4 expression in cattle-yak and yak spermatogenic cells cultured in vitro, respectively. The expression of β-actin was served as control

The spermatogenic cells of cattle-yak and yak were sub-cultured three times in vitro and then collected for proliferation, viability and signature gene expression analysis. We found that the proliferation rate of yak spermatogenic cells was higher than that of cattle-yak (Fig. S4A and S4B; Fig. 4A). Compared with yak, the SSC marker gene (*THY1*)*,* differentiating SPG marker (*UCHL1, c-kit, STR8*) and meiosis recombination marker (*SYCP3*) were downregulated in yak spermatogenic cells sub-cultured three times in vitro, while SSC marker gene (*GFRA1*) and differentiating SPG markers (*KI67*) were upregulated in cattle-yak (Fig. 4B). On the protein level, PLZF exhibited higher expression in cattle-yak, while GFRA1 showed equivalent expression level in cattle-yak and yak. In accordance with gene expression, the c-kit was barely expressed in cattle-yak (Fig. 4C). Therefore, the spermatogenesis of cattle-yak probably arrested at pre-meiotic or even SPG differentiation stage.

### Comparative of spermatogonial cohorts and further analysis of proliferation potentials of UDSPG between cattle-yak and yak in vitro

**Fig. 5:**
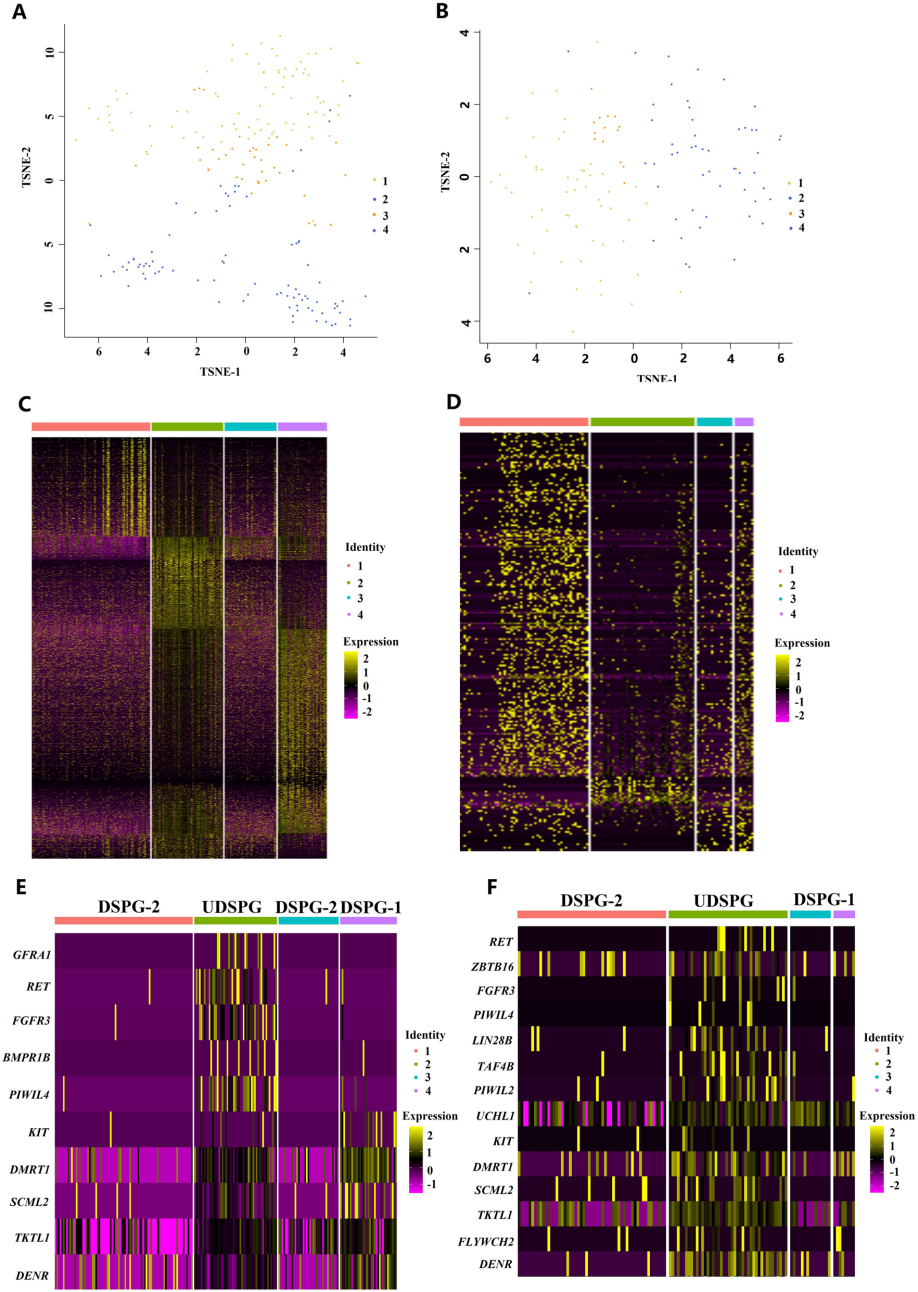
Re-clustering of spermatogonia and marker gene expression analysis in sub-clusters. **A**,** B** tSNE plots of SPG identified in cattle-yak and yak, respectively. **C**,** D** Heatmap of DEGs in four sub-clusters of SPG in cattle-yak and yak, respectively. The color gradient from yellow to purple shows high to low expression levels. **E, F** Heatmap of some marker gene expressions in the sub-clusters of SPG in cattle-yak and yak, respectively. The color gradient from yellow to purple shows high to low expression levels

To characterize SPG cohorts in different developmental stages, we re-clustered Cluster 4 in cattle-yak and Cluster 7 in yak. Both analyses yielded four sub-clusters and those from yak exhibited higher similarity (Fig. 5A-D). The four sub-clusters from both species were further identified as UDSPG, differentiating SPG (DSPG-1) and differentiated SPG (DSPG-2) based on their comprehensive marker gene expression analyses (Fig. 5E and F). The known SSC markers (*GFRA1*, *BMPR1B, RET, FGFR3* and *PIWIL4*) were exclusively or highly expressed in UDSPG of the two species. Additionally, higher expressions of *LIN28B*, *TAF4B*, *PIWIL2* and *UCHL1* were also examined in UDSPG of yak (Fig. 5F), in which *TAF4B* is required for UDSPG development and *PIWIL2* for UDSPG self-renewal [49, 50]. The differentiating makers, *UCHL1*, *KIT* and *DMRT1* were highly expressed in cattle-yak and yak DSPG-1, while *SCML2*, *TKTL1*, *FLYWCH2* and *DENR* showed high expressions in DSPG-2 [21, 51].

**Fig. 6:**
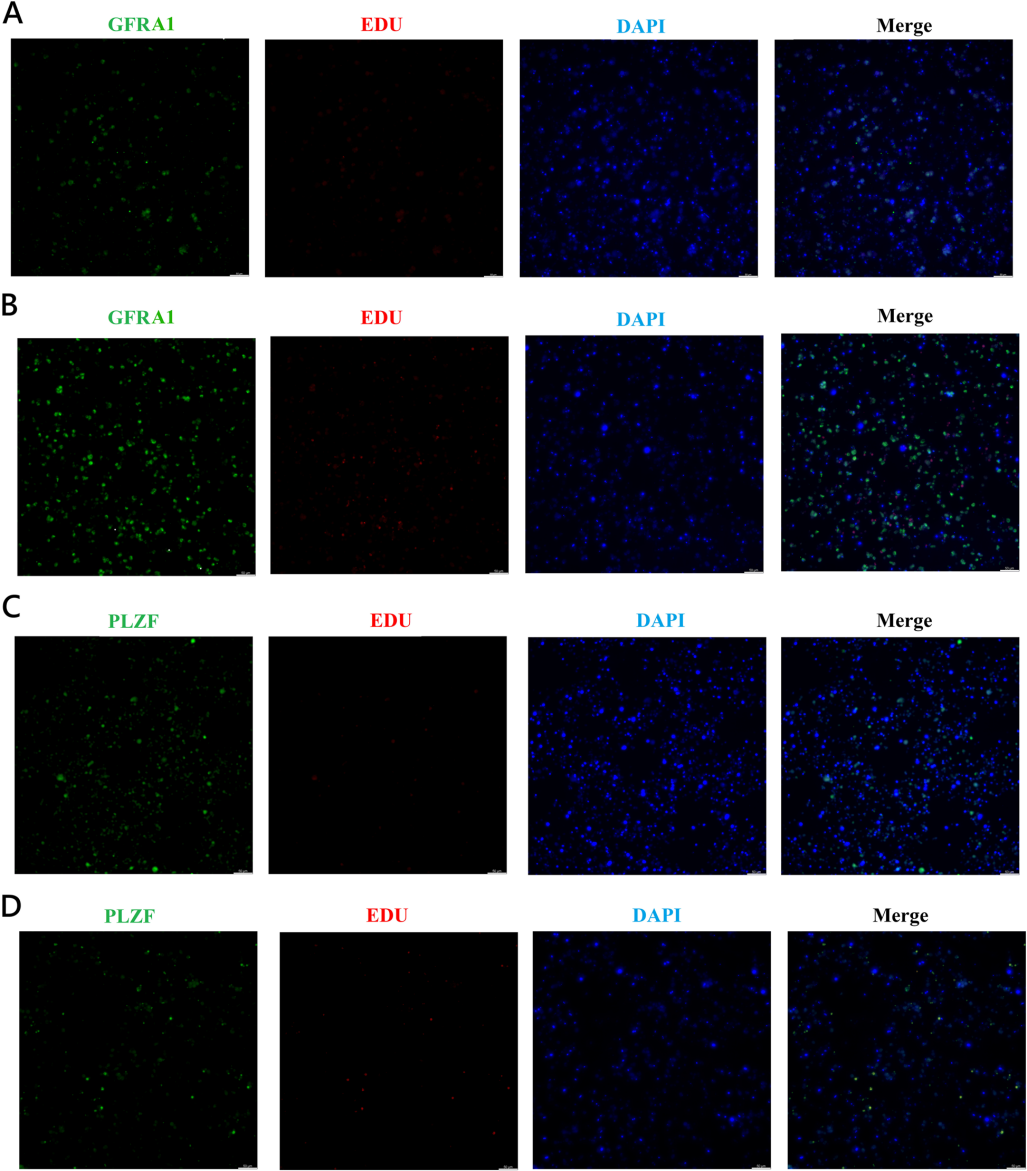
The proliferation of UDSPG in spermatogenic cells sub-cultured three times in vitro. **A, B** Immunofluorescence of GFRA1 (green) co-stained with EDU (red) on UDSPG of cattle-yak and yak after co-cultured with their own testis somatic cells, respectively. **C, D** Immunofluorescence of PLZF (green) co-stained with EDU (red) on UDSPG of cattle-yak and yak after co-cultured with their own testis somatic cells, respectively. The scale bars represent 50 μm

We found that spermatogenic cells of cattle-yak and yak co-cultured with their own testis somatic cells presented larger cell clones and higher viability (Fig. S5A-S5D). Then, we identified UDSPG of cattle-yak and yak from spermatogenic cells by immunofluorescence method after co-cultured with their own testis somatic cells and sub-cultured three times in vitro. Higher proportion of GFRA1^+^ UDSPG was identified from cattle-yak spermatogenic cells, while more PLZF^+^ UDSPG presented in yak (Fig. 6A-D). In addition, proliferation analysis of GFRA1^+^ and PLZF^+^ UDSPG co-stained with EDU revealed that both GFRA1^+^ and PLZF^+^ UDSPG of yak presented higher proliferation activity than those of yak (Fig. 6A-D). Subsequently, we induced these spermatogenic cells of cattle-yak and yak with retinoic acid (RA) in vitro to further investigate their differentiation potentials. RA stimulation resulted in the expression of *STR8* and *c-kit* in both cattle-yak and yak spermatogenic cells (Fig. S6A). *BOLL, DAZL* and *SYCP3* were upregulated in yak spermatogenic cells, while they were barely expressed in cattle-yak (Fig. S6A). In addition, the percentage of spermatocytes of yak was increased after AR inducion, while no increase of spermatocytes was observed in cattle-yak (Fig. S6B-S6D). Western Blotting analysis revealed the SYCP3 expression in yak after AR inducion, but not in cattle-yak (Fig. S6E). In summary, these results indicated that UDSPG of cattle-yak presented lower proliferation and differentiation potentials.

### Comparative analysis of niche cell cohorts between cattle-yak and yak

**Fig. 7:**
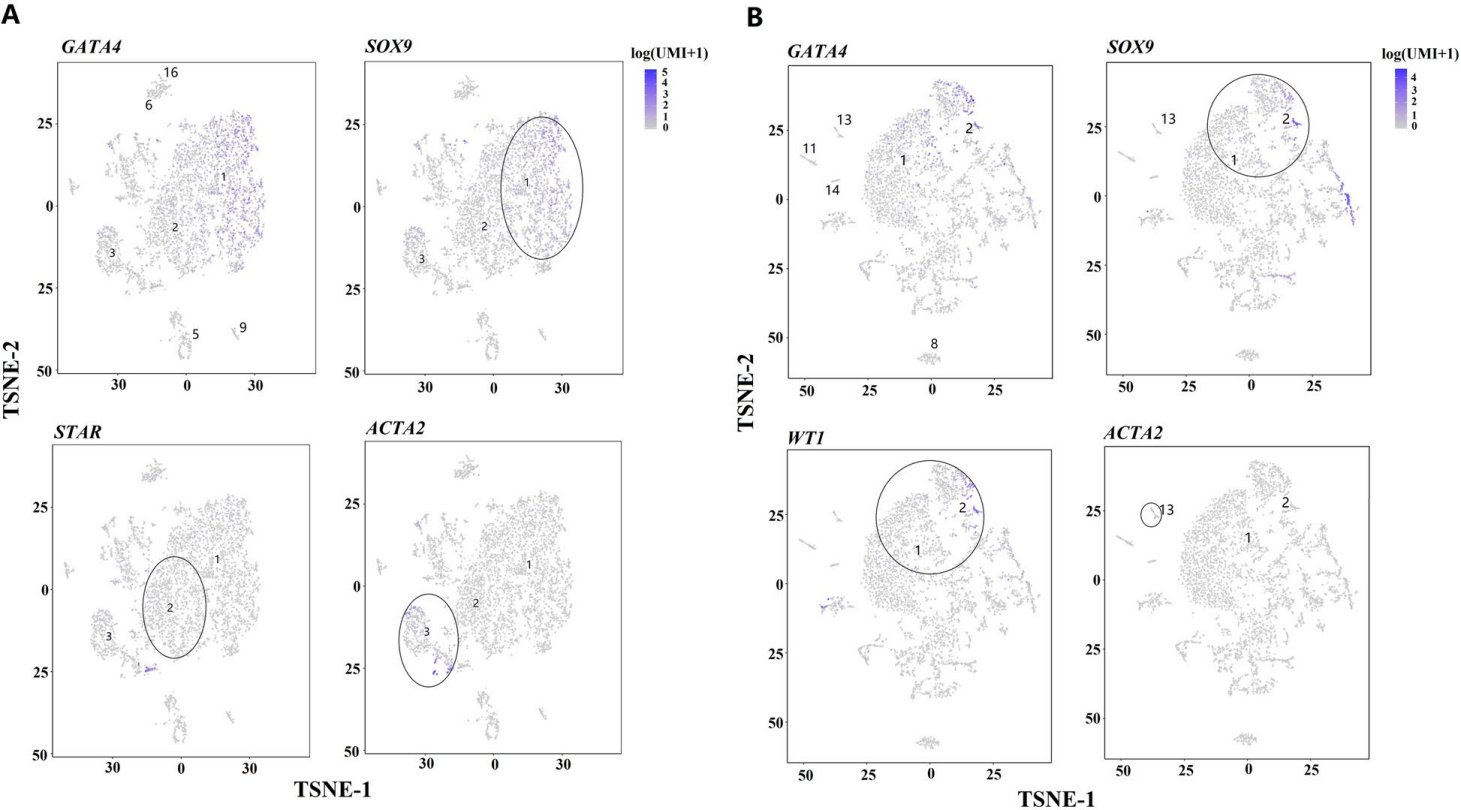
Analysis of niche cell cohorts of cattle-yak and yak their proliferation in vitro. **A, B** Expression patterns of selected niche cell marker genes on tSNE plots of cattle-yak and yak, respectively. The blue gradients from dark to light indicate high to low expression levels

Niche cells account for a larger proportion in pubertal cattle-yak and yak testicular cells and more niche cells (marked by *GATA4*) were identified in cattle-yak (Fig. 2A and B; Fig. 7A and B). Although Sertoli (marked by *SOX9*) and Leydig cells (marked by *STAR*) were the main components of niche cells in both cattle-yak and yak, much more peritubular (marked by *ACTA2*) and perivascular cells were identified in cattle-yak (Fig. 2A and B; Fig. 7A and B).

## Discussion

The development of scRNA-seq analyses currently enabled us to identify nearly all heterogeneous cell types in high-resolution from seemingly homogeneous tissues and also the dynamic gene expression patterns of cells in different developmental stages. Both scRNA-seq data and global composition analysis revealed that cattle-yak testicular cells presented the defects in lower density, smaller size and lower proportion of DDX4^+^ germ cells in comparison with yak, which was in accordance with the testis tissue and cellular characteristics of cattle-yak examined previously [15, 16, 26]. Notably, more than 95% of the testicular cells in pubertal cattle-yak were identified to be niche cells, and about half of the testicular cells were niche cells in yak, the percentages of which was dominantly lager than that identified from pubertal goats [23]. These results could be attributed to the harsh living and nutritional environments of yak on Qinghai-Tibet plateau and developmental delay compared to other large domestic animals fed in lower altitude regions. The “testicular puberty” of cattle-yak was obviously not fully triggered and the proportion of niche cells was approximate to that identified from perinatal period testis [24, 52].

Meiotic entry characterized by emerging of pachytene spermatocytes is crucial for the procession of spermatogenesis and the lower efficiency or failure of meiotic entry was observed in azoospermic patients [53, 54]. The spermatogenesis of cattle-yak was apparently arrested at pre-meiotic development stage for the number of late pri. SPC were sharply reduced at meiotic entry stage, while the successive developmental trajectory of germ cells for SPG, early pri. SPC, late pri. SPC, Diak. To sec. SPC, early SPT and Late SPT were presented and no reduced numbers of late pri. SPC were found in yak. Seemingly, these results convinced the generally accepted view that MHS of cattle-yak was caused by meiotic arrest at about the pachytene stage [8, 11, 12]. However, in vitro culture and differentiation analysis of testicular cells revealed that cattle-yak spermatogenic cells presented lower proliferation potentials, which confirmed the findings from comparative testis transcriptome data that spermatogenic arrest of cattle-yak may originate from the stage of spermatogonial differentiation [14]. Thus, our data on the germ cell atlas and their developmental landscapes provided a more clear view on spermatogenic arrest of Cattle-yak.

To further examine the components and differentiation potentials of spermatogonia between cattle-yak and yak, we re-clustered SPG populations and identified sub-clusters of UDSPG, DSPG-1 and DSPG-2. Both cattle-yak and yak UDSPG exhibited the specific expressions of known such SSC marker genes as *GFRA1*, *RET*, *ZBTB16 (PLZF)*, *FGFR3*, *BMPR1B* and *PIWIL4*, which were common SSC markers in human and mouse [20, 21, 55]. Among these markers, only *GFRA1* and *ZBTB16 (PLZF)* were found to be specifically expressed in cattle bulls, and other markers may also be used to identify and isolate UDSPG in the bovid species as cattle (*Bos taurus*), goat (*Capra hircus*) and sheep (*Ovis aries*) [25, 56, 57]. In the spermatogenic cells of cattle-yak and yak co-cultured with their own testis somatic cells, we identified higher proportion of GFRA1^+^ UDSPG in cattle-yak and higher proportion of PLZF^+^ UDSPG in yak, which was in accordant with the *GFRA1* and *ZBTB16 (PLZF)* expressions in scRNA-seq data. Moreover, both GFRA1^+^ and PLZF^+^ UDSPG of cattle-yak presented lower proliferation activity and differentiation potentials than those of yak, which confirmed our previous findings based on comparative testis transcriptome analyses that spermatogenic arrest of cattle-yak may originate from the differentiation stage of spermatogonial stem cells and be aggravated during spermatogonial mitosis and spermatocyte meiosis [14].

Niche cells provide the specialized microenvironment for spermatogenesis in mammals [58]. In pubertal cattle-yak and yak, we found that Sertoli and Leydig cells were the main components of niche cells, while much more peritubular and perivascular cells were identified in cattle-yak. Therefore, the abnormity in components and proliferation of niche cells may also contribute to MHS of cattle-yak. Sertoli cells are the key supportive cells for spermatogenesis and the main controller of microenvironment within the seminiferous tubules [59]. In accordance to the testis transcriptomic and proteomic data, the elevation of protein synthesis in Sertoli cells would give rise to the upregulation of these inter-germ-cellular genes and proteins, which may prevent germ cell migrating towards the seminiferous tubule lumen and further contributed to MHS of cattle-yak [14, 15]. Additionally, increased expression of intercellular matrix genes and proteins from peritubular and perivascular cells may be the causes of testicular interstitial fibrosis in cattle-yak [14, 15].

## Conclusions

Comparative scRNA-seq analysis in this work indicated that dynamic gene expression of developmental germ cells was terminated at late pri. SPC (meiotic arrest) and abnormal components of niche cell in pubertal cattle-yak. And further in vitro proliferation revealed more causes than meiotic arrest contributed to MHS of cattle-yak. UDSPG of cattle-yak exhibited defects in viability and proliferation/differentiation potentials, which confirmed our previous conclusions that spermatogenic arrest of cattle-yak may originate from the differentiation stage of spermatogonial stem cells and niche cells of cattle-yak may provide an adverse microenvironment for spermatogenesis. However, current data in this work is far from enough to unveil the mechanism underlying MHS of cattle-yak, further work could be carried to investigate the genomic component/function divergences between cattle-yak and their paternal species should also be explored in the future studies.

## Methods

### Experimental animals, tissue sample collection and tissue cryopreservation

Three pubertal cattle-yaks (♂ cattle × ♀ Maiwa yak) and two pubertal yaks (Maiwa breed) aged 24 months were obtained from a private pasture in Hongyuan county, Sichuan province, China. These animals were fed on the same pasture. The cattle-yaks and yaks had a mean weight of 110 and 90 kg, respectively. Twenty minutes before castration by veterinary surgical operation, the animals were caught and 3 ml of local anaesthetic (Lignocaine 2%, Chengdu Mainpines Biological Co., Ltd., China) was injected into the opposite pole of each testicle and the anesthetic was distributed by massaging the scrotum. Castration was carried out as for each animal 20 min after local anaesthetic and the testis of each animal was obtained by using an elastrator (Guangxi Jiangs Animal Product Co., Ltd., China). After the wound of each scrotum was sprayed with a chlorhexadine solution (20%), the animal was released, fed and cared individually. Subsequently, epididymis, fat and fascia tissue were removed from each testis and three slices of testicular samples were crosscut from the middle of testis by fine scale dissection. Then, the testis samples were transferred to lab within 30 min. After the three slices of samples from each testis were mixed together and cut into cut into small pieces with the volume of 6 mm^3^, a little part of sample from each testis was used for preparation of single cell solutions. A large part of each testis sample was washed with PBS solution supplemented with 100 mg/mL streptomycin and 100 IU/mL penicillin, and was used for cryopreservation and cell culture experiment. The tissues were soaked with cryoprotective solution (10% DMSO, 10% fetal bovine serum, 80% complete medium), incubated 5 min at room temperature and then stored in liquid nitrogen. The frozen tissues were transferred to laboratory for the following experiments.

### Preparation of testicular single cell solutions

The testis samples from each individual were cut into smaller pieces with the volume less than 0.5 mm^3^ after washing three times with PBS, then subjected to enzymatic digestion comprising Collagenase IV (1 mg/mL) and Dnase I (1 μg/μL) in a 1.5 mL centrifuge tube at 37 °C for 15 min as described previously [16, 26]. During the digestion period, the tissues were mixed by pipetting up and down once in every five minutes. Enzymic digestion was terminated by adding 10% FBS (in DMEM) and testicular single cells were obtained by filtering through a 40-μm nylon mesh. After centrifugation at 1500 rpm for 5 min at 4 °C, the supernatant material was removed and the single cells were washed with 0.04% BSA DPBS for three times and the cell number and cell viability were measured by using the Countess® II Automated Cell Counter. The cells were resuscitated to a concentration of 700–1200 cells/μL with a viability ≥ 85%, ready for scRNA-seq.

### ScRNA-seq performance, cDNA library construction and sequencing

Single-cell RNA-seq was performed by employing 10 × Genomics system. In brief, the GEMs (Gel Beads-in-emulsion) generation (combining barcoded Single Cell 3ʹ v3 Gel Beads, a Master Mix containing cells and Partitioning Oil onto Chromium Chip B) was followed by the dissolution of Gel Bead, release of primers and lysation of co-partitioned cell. As a result, the primers comprising an Illumina TruSeq Read 1, 16 nt 10 × Barcode, 12 nt unique molecular identifier (UMI) and 30 nt poly (dT) sequence were mixed with the cell lysate and a Master Mix containing reverse transcription (RT) reagents. Subsequent incubation of the GEMs produced barcoded full-length cDNA from poly-adenylated mRNA, which was amplified via PCR to generate sufficient mass for 3ʹ gene expression library construction. A total of 50 ng of amplified cDNA was fragmented and end-repaired, double-size selected with SPRIselect beads and sequenced on a NovaSeq platform (Illumia) to generate 150 bp paired-end Reads.

### Transcriptome alignment, cell clustering and cell type identification

Raw data were assembled from the Raw BCL files using Illumina’s bcl2fastq converter, processed through primary quality control and the clean data were used for downstream analyses. STAR software implemented in Cell Ranger was used to align the Reads to the reference genome of *Bos grunniens* (assembly BosGru3.0), and Cell Ranger software 3.0 was used for the transcript annotation GTF to bucket the Reads into exonic, intronic and intergenic region of the genome. Reads that were confidently mapped to the transcriptome are placed into groups that share the same barcode, UMI, and gene annotation. Principal Components Analysis (PCA) implemented in Cell Ranger was employed to change the dimensionality of the dataset from (cells × genes) to (cells × M), where M is a user-selectable number of principal components. A python implementation of IRLBA algorithm was modified to reduce memory consumption. PCA-reduced data were passed into tSNE and could be visualized in two-dimensional space. K-means clustering was used to cluster cells across a range of K values (2–35), where K is the preset number of clusters. The default selected value of K is that which yields the best Davies-Bouldin Index, a rough measure of clustering quality. After analyzing the resulted cell clusters by using the known marker genes on by one, the testicular cells from both cattle-yak and yak could be distinguished from each other when clustered into 17 clusters. The DEGs between cell cluster was determined by the mean UMI > 1 and the fold change calculated by comparing mean counts between each cluster and the means of all clusters. The upregulated genes were identified by log_2_ fold change > 0 and the downregulated by log_2_ fold change < 0 (*P* < 0.05). To further identify UDSPG, DSPG-1 and SPG-2, Cluster 4 representing SPG of cattle-yak and Cluster 7 representing SPG of yak were re-clustered to generate four sub-clusters, respectively.

### Cell trajectory analysis

Single cell pseudotime trajectories for germ cells were constructed by Monocle based on the differentially expressed marker genes of different germ cells (Table 1). The heatmaps and dot plots were also generated in accordant with developmental order of germ cells in pseudotime trajectories.

**Table 1:** The differentially expressed marker genes of different germ cells used for construction of single cell pseudotime trajectories

Bovid species	Germ cell types	Marker genes
Cattle-yak	SPG	*RET*, *DMRT1*, *TKTL1*
	early pri. SPC	*SPO11*, *MEIOB*, *RAD51AP2*
	late pri. SPC	*ADAM2*, *NME8*, *CCNA1*
Yak	SPG	*RET*, *PIWIL4*, *UCHL1*
	early pri. SPC	*RAD51AP2*, *SYCP1*, *MEIOB*
	late pri. SPC	*NME8*, *CCNA1*, *SPATA16*
	Diak. To sec. SPC	*TMEM89*, *CCDC81*, *OLFML2B*
	early SPT	*KLF5*, *TJP3*, *ACTRT3*
	late SPT	*TNP1*, *SPEM1*, *GAPDHS*

*SPG* Spermatogonia, *pri. SPC* Primary spermatocytes, *Diak. To sec. SPC* Diakinesis to secondary spermatocytes, *SPT* Spermatids

### Isolation and culture of spermatogenic cells from cattle-yak and yak

The frozen testicular tissue (0.5 g) was taken out from liquid nitrogen and incubated at 37 ℃ for few minutes until the tissue was thawed. Then, the testicular tissue was immersed in the medium (DMEM/F12 + 0.5 mol/L sucrose + 20% FBS) at 37 ℃ for 2 min. After washed with PBS for 2–3 times, the tissue was cut into pieces with eye scissors followed by enzymic digestion (1 mg/mL collagenase Type IV + 0.5 μg/mL DNase I) in the water bath shaker at 37 ℃ for 30–40 min. The enzymic digestion was terminated by adding 10% FBS (in DMEM). The mixture was filtered with 70 μm and 40 μm sieve and centrifuged at 300 g for 5 min at 4 ℃. The supernatant was discarded and the cells were re-suspended in 10% FBS medium, and then cultured in vitro at 37 ℃, 5% CO_2_. After cultured for 24 h, the suspension cells were carefully collected by centrifugation at 300 g for 5 min and the cell pellet was re-suspended with DMEM high glucose medium (5% KSR, 5% FBS, 20 ng/μL GDNF, 20 ng/μL EGF, 1% glutamine, 1% non-essential amino acids and 1% antibiotic) and cultured for another 24 h. During the period of culture, the suspension cells were collected again by differential adhesion and observed under the microscope.

### The proliferation of spermatogenic cells from cattle-yak and yak

The spermatogenic cells sub-cultured three times were fixed with 4% paraformaldehyde for 20 min at room temperature, and 50 μL of cell suspension was dropped onto the adhesive slide. After evaporated at room temperature for 30 min, spermatogenic cells on the slide was blocked with 3% BSA at room temperature for 1 h and incubated with the DDX4 antibody (1:250, Santa Cruz) overnight at 4 °C. The next day, the slide was incubated with the secondary antibody at room temperature for 2 h, then the nucleus was stained with DAPI (5 μg/mL) for 5 min. The images were recorded by microscope (Leica, DMI9). Spermatogenic cells (1.0 × 10^4^) sub-cultured three times were planted to 96-well plate. Then, the cells (10μL) was added into each well and incubated for 4 h. The absorbance of different groups was detected at 450 nm and the relative survival rate was analyzed, and the proliferation potential of spermatogenic cells was detected by using EdU method. The cells was fixed with 4% paraformaldehyde for 30 min, neutralized with 2 mg/mL glycine, washed with PBS and then planted on the adhesive slide. After permeated with 0.5% triton X-100 (100 μL) for 10 min, the cells on the slide was stained with EdU for 30 min (100 μL). The nuclei were stained for 30 min, and then the images were recorded by microscope. Meanwhile, spermatogenic cells were collected for marker gene (*GFRA1, THY1, UCHL1, KI67, c-kit, STR8* and *SYCP3*) and protein expression (PLZF, GFRA1, c-kit and DDX4) analysis by qRT-PCR and Western blotting, respectively.

### Identification and proliferation analysis of UDSPG from cattle-yak and yak.

After fixed with 4% paraformaldehyde, the spermatogenic cells were patched to slide. The slide was washed with PBS and the cells were blocked with 3% BSA at room temperature for 1 h. Subsequently, the slide was incubated with primary antibody GFRα1 (1:200, Abcam) and PLZF (1:200, Santa Cruz) at 4 °C overnight. The next day, the slide was incubated with the secondary antibody at room temperature for 2 h, then the nucleus was stained with DAPI (5 μg/mL) for 5 min. The images were recorded by microscope. After EDU staining as described above, spermatogenic cells were blocked with 3% BSA for 1 h at room temperature. Then, GFRα1 and PLZF immunofluorescence staining was performed as above and the images were recorded by microscope.

### Differentiation potentials of spermatogenic cells of cattle-yak and yak co-cultured with their own testicular somatic cells

Enzyme digested testicular tissue cells were divided into two parts after differential adhesion and the adhered cells were somatic cells. Somatic cells were planted in a density of 10% as feeder and spermatogenic cells (1.0 × 10^5^) of cattle-yak and yak were planted to 12-well plates to co-culture with somatic cells, respectively. The growth status of spermatogenic cells was recorded by microscope, and spermatogenic cells were collected for marker gene and protein expression analysis as previously. Spermatogenic cells sub-cultured three times were seeded in 12-well plates with 1.0 × 10^5^ cells in each well and the culture medium was replaced by inducion medium (5% KSR, 5% FBS, 20 ng/μL GDNF, 20 ng/μL EGF, 1% glutamine, 1% non-essential amino acids, 30 ng/μL retinoic acid (RA) and 1% antibiotic in DMEM high glucose medium). The control group was cultured in the medium without RA, which was replaced by the same volume of DMSO. After 5 days of inducion, the spermatogenic cells were collected for relative expression level detection of mRNA (*STR8, c-kit, Boll, DAZL* and *SCYP3*) and protein (SYCP3). The particle size and distribution of spermatogenic cells in each group were detected using an automatic cell counter (JIMBIO, China).

### Reverse transcription quantitative real-time PCR

Total RNA was extracted from testicular tissues or cells by Trizol method (15,596,018, Ambion) and reverse transcription was performed using Takara Reverse Transcription Kit (RR047A). RT-PCR was performed according to instructions of HISCRIPT III RT Super Mix for QPCR (R323-01, Nazyme), and the expression levels of different genes were detected with quantitative PCR amplification method by using CHAmQ Universal SYBR QPCR Master Mix (Q711, Nazyme). Each reaction volume (20 μL) comprised 0.3 ng cDNA template and 300 pmol of primers. Each PCR reaction was performed in triplicate on CFX96 Touch™ Real-Time PCR Detection System (BIO-RAD), using the program as follows: 95 °C, 3 min; 40 cycles of 95˚C for 10 s and 60 ˚C for 1 min. Fold change of gene expression was calculated using the 2^−ΔΔct^ method and was expressed as a ratio of expression levels of treated groups to the expression level of the control group. The primer sequences for validation of cell type specific gene expressions in testicular cells between cattleyak and yak are shown in Table S4, and for detection of germ cell mark genes during spermatogenic cell proliferation & differentiation are shown in Table S5.

### Western blotting

The protein lysis buffer (Beyotime, P0013B, China) containing protease inhibitors (Roche, 11,697,498,001, Switzerland) was added to lysate the spermatogenic cells, and incubated on ice for 30 min. The lytic mix liquids were centrifugated at 12,000 × g for 15 min to collect the supernatants. The protein was quantified with BCA method (Beyotime, P0012S, China), then mixed with 5 × loading buffer (Beyotime, P0015, China) and denatured at 95℃ for about 10 min. Subsequently, 15 μg of total protein and 5 μL of protein Marker (P0068, Beyotime) was added to 10% SDS-PAGE electrophoresis gel (Beyotime, P0690, China), respectively. Electrophoresis was performed at 90 V and the voltage was adjusted to 120 V when the Marker ran past the concentrated gel, and then the protein was transferred to PVDF membrane. After blocked with 5% nonfat-dried milk buffer, primary antibody (PLZF/GFRA1/β-actin/SYCP3/DDX4/c-Kit) was added to change the blocking buffer and incubated overnight at 4℃. HRP-labeled secondary antibody was incubated with the membrane at room temperature in an hour, and then the ECL Chemiluminescent substrate (4A BIOTECH, w011-20, China) was added to cover the PVDF membrane. Finally, the image was recorded with chemiluminescence imager (ChemiDoc Touch, Bio-Rad).

### Statistics and analysis

Statistical analysis was performed by using GraphPad Prism software. Each group of experiments was repeated more than 3 times, and t-test was used for significance analysis. *P* < 0.01 means the difference is extremely significant and *P* < 0.05 means the difference is significant.

## Supplementary Information

**Additional file 1: Table S1.** Statistics of cell numbers, scRNA sequencing and transcriptome alignment.

**Additional file 2:** **Table S2.** DEGs between the identified 17 testicular cell clusters in cattle-yak.

**Additional file 3:** **Table S3.** DEGs between the identified 17 testicular cell clusters in yak.

**Additional file 4:** **Table S4.** Primer sequences for validation of cell type specific gene expressions in testicular cells between cattle-yak and yak.

**Additional file 5:** **Table S5.** Primer sequences for detection of germ cell marker genes during spermatogenic cell proliferation and differentiation between cattle-yak and yak.

**Additional file 6:** **Fig. S1.** Identification of pubertal testicular cells from cattleyak and yak. (A and B) The re-suspension cells of cattleyak and yak after 2 days of culture, respectively. (C and D) The size and distribution of testicular cells from cattleyak and yak, respectively. (E and F) Immunofluorescence of DDX4 (green) in pubertal testicular cells of cattleyak and yak, respectively. The scale bars represent 50 μm. **Fig. S2.** Validation of cell type specific gene expressions between testicular cells of cattleyak and yak. (A) Log10 fold change values for RT-qPCR detection of 46 germ cell specific signature gene expression in pubertal testes between cattleyak and yak. (B) Log10 fold change values for RT-qPCR detection of 10 niche cell type specific signature gene expression in pubertal testes between cattleyak and yak. YK and CY denote yak and cattleyak, respectively. * indicates the differentially expressed signature genes in undifferentiated spermatogonial cells. **Fig. S3.** Dot plot showing relative expression patterns of the potential marker genes for each germ cell type (A) Relative expression of the potential marker genes for spermatogenic cells in cattleyak. (B) Relative expression of the potential marker genes for spermatogenic cells in yak. **Fig. S4.** Proliferation analysis of the spermatogenic cells cultured in vitro by EDU staining. (A, B) EDU and DAPI staining of cattle-yak and yak spermatogenic cells, respectively. **Fig. S5.** Spermatogenic cells co-cultured with different testis somatic cells and cell viability analysis. (A) Yak spermatogenic cells co-cultured with yak testicular somatic cells as feeder and sub-cultured three times in vitro. (B) Cattleyak spermatogenic cells co-cultured with cattleyak testicular somatic cells as feeder and sub-cultured three times in vitro. (C) Cattleyak spermatogenic cells co-cultured with yak testicular somatic cells as feeder and sub-cultured three times in vitro. (D) CCK8 assay detected the viability of yak and cattleyak spermatogenic cells co-cultured with their testicular somatic cells for 4 consecutive days. The scale bars represent 50 μm. * denotes P<0.05, and *** denotes P<0.001. **Fig. S6.** Inducion of spermatogenic cells of cattleyak and yak with retinoic acid (RA) in vitro. (A) STR8, c-kit, BOLL, DAZL and SCYP3 mRNA expressions in of spermatogenic cells of cattleyak and yak after AR inducion. (B and C) The cell size and distribution of spermatogenic cells after AR induction in yak and cattle yak, respectively. (D) Percentage of spermatocytes of spermatogenic cells after AR inducion in yak and cattle yak, respectively. (E) SYCP3 protein expression in spermatogenic cells after AR induction in yak and cattleyak, respectively.

## Data Availability

The raw data of single cell RNA-seq are available from Genome Sequence Archive (GSA) under accession CRA006268 in CNCB (China National Center for Bioinformation) (https://bigd.big.ac.cn/gsa/browse/CRA006268). All data are available in the main text or the supplementary materials.

## Abbreviations

CY: Cattle-yak
YK: Yak
MHS: Male hybrid sterility
DEG: Differentially expressed gene
scRNA-seq: Single-cell RNA sequencing
UDSPG: Undifferentiated spermatogonia
DSPG: Differentiated spermatogonia
SPG: Spermatogonia
pri. SPC: Primary spermatocytes
SC: Synaptonemal complex
Diak. To sec. SPC: Diakinesis to secondary spermatocytes
SPT: Spermatids
DSPG-1: Differentiating SPG
DSPG-2: Differentiated SPG
tSNE: T-distributed stochastic neighbor embedding
RA: Retinoic acid
FACS: Fluorescence-activated cell sorting
PCA: Principal components analysis
