# Supplementary material for: Single-cell transcriptome analysis and in vitro differentiation of testicular cells reveal novel insights into male sterility of the interspecific hybrid cattle-yak
Source: BMC Genomics. 2023 Mar 27;24:149. doi: 10.1186/s12864-023-09251-2 (PMC10045231; doi:10.1186/s12864-023-09251-2)
Supplement: Supplementary file 1 — Additional file 1: Table S1. Statistics of cell numbers, scRNA sequencing and transcriptome alignment. [file 12864_2023_9251_MOESM1_ESM.docx]

**Table S1. Statistics of cell numbers, scRNA sequencing and transcriptome alignment**

| Samples | Cell numbers | Mapping rate | Sequencing saturation rate | Gene numbers per cell | Median genes per cell | Mean reads per cell |
| --- | --- | --- | --- | --- | --- | --- |
| CY1 | 1339 | 92.5% | 80.1% | 17628 | 867 | 153266 |
| CY2 | 3389 | 92.9% | 92.6% | 15936 | 1001 | 352053 |
| CY3 | 1401 | 93.0% | 90.8% | 16075 | 1016 | 311330 |
| YK1 | 2598 | 93.5% | 83.3% | 18628 | 1137 | 297974 |
| YK2 | 1261 | 92.7% | 72.3% | 18389 | 700 | 117866 |
| Total | 9988 | / | / | 86656 | 4721 | 1132489 |
| Average | 1998 | 92.9% | 83.8% | 17331 | 944 | 246,498 |
