# Supplementary material for: Single-cell transcriptome analysis and in vitro differentiation of testicular cells reveal novel insights into male sterility of the interspecific hybrid cattle-yak
Source: BMC Genomics. 2023 Mar 27;24:149. doi: 10.1186/s12864-023-09251-2 (PMC10045231; doi:10.1186/s12864-023-09251-2)
Supplement: Supplementary file 4 — Additional file 4: Table S4. Primer sequences for validation of cell type specific gene expressions in testicular cells between cattle-yak and yak. [file 12864_2023_9251_MOESM4_ESM.docx]

**Table S4 The primer sequences for validation of cell type specific gene expressions in testicular cells between cattle-yak and yak**

| Genes | Primer sequence(5′→3′) |
| --- | --- |
| *PLZF* | F: GTTCCATGCCCACCGGACT R: TGGCCTCCGTGTCATTGTCA |
| *RET* | F: CTCACGAGGGATGCAGTACC R: GGTCACAATCTCCCACAGCA |
| *PIWIL4* | F: GGCGCTGAACAGGTGGTATA R: GCCCTTCTTCCTGACCACAA |
| *TAF4B* | F: TCAGTGAAGAAAACGCCTGC R: TCCTGTGTTGCATGGGAGAT |
| *FGFR3* | F: TCTGGCGGGCAATTCTATCG R: GGTGTGTTGGAGCTCATGGA |
| *LIN28B* | F: ACAACTGTGGTGGCCTTGAT R: CGCTTCCTGTCTTCCCTGAG |
| *SCML2* | F: AACATGGAAACTGTGGCCCT R: CTCCTCCACGATCATCTGGC |
| *TKTL1* | F: AGCCCCTATTGAAGACTCGC R: CACGCTCACCATGTTTTGCT |
| *DMRT1* | F: CCCGTGCCTGATGATTGAGA R: GTACGGAAACAGAGACGGCT |
| *SYCE1* | F: GAGAAGCTGGTGGAGGCAAA R: GGGCTTTCCTGTTCTCCTCC |
| *SPO11* | F: TCGAAGCCCATAATCTCACA R: CTGCCTTCATTTTAGAGTCTG |
| *TOP2B* | F: AAAAGGTAAAGGCCGAGGGG R: TGAGGTCTGAGGGGAAGAGG |
| *DMRTC2* | F: TCTTTCAGGCATGCGAGTGT R: GACTCCCTTCTTGACACGGC |
| *FLYWCH2* | F: TCCAAGGACAGTGCCAAAGTG R: GCCCTGCTAAGCTCCCATC |
| *IFT81* | F: TCCAGGAAATGCTGCAGACA R: TCAGCCACGGTTTCATCCTG |
| *RAD51AP2* | F: ACGCCTCTGTACACGATGTA R: CCGCGCTGGTTTTGAGATTT |
| *DENR* | F: GAATATATGCCTGATGTTGC R: TCTGTTTTATTTGACCCCTT |
| *ORC6* | F: CAGTCTTCCACAAACACAGCA R: CGATCTGCTGCCCAATCTTT |
| *SYCP2* | F: AGTGGAAAGTTTCGTGCCTC R: GATACCCGCTTGCAAGTCAT |
| *MLH1* | F: GGTCGTTAAAGAGGGAGGCC R: CTGCACACGGTTTAGGAGGT |
| *ACRBP* | F: GTACCCCAACTACTGTGCCT R: AAACTCCTGGCTCCATCGAA |
| *AURKA* | F: GGAATATGCACCCCTCGGAG R: GGCATGTACTGACCACCCAA |
| *ZPBP2* | F: ACGTATGGATAGCTGTCGTCC R: ACTGGCATGATTTAGCTCCGT |
| *NME8* | F: AGACCAGCCTGACTTTGAGG R: GCTTGGCCATCTCCTGTTGT |
| *CCDC42* | F: CTGGCACGCTACAAAGAGGA R: AGGTTCAGCGTTGCCATCTT |
| *C9orf116* | F: GCCGGAGAAAACCAGCGACT R: CTTGGTCCTGTAGCCCCGAA |
| *CIB4* | F: TCCGGCTGCTCAATAGTGAC R: GCCATTGCGTGTTCAAACTCT |
| *TMEM89* | F: CTGGAGGGTTGTGAGAGCAG R: TCTTAGAGGCCTGTGAACGC |
| *OLFML2B* | F: ATCACAGGGCCGATTACACA R: CCCGGTTGTAGTAGAAGGCA |
| *C11orf97* | F: ATGCCACATTAAACATCCACC R: TGCCTTGAATAGTATTTGGCT |
| *CCDC81* | F: GCTGTCTCTTCTGCCAAAGG R: TGTCCTGCCCTATGATGGTC |
| *C9orf135* | F: TGCCGTTCTCAGTTCACAGA R: AAAAGGGAGCAATAGGGCCA |
| *TJP3* | F: GACTACGAGACAGATGGCGA R: TGTCCGCATGCTTTCTTGTC |
| *ACRV1* | F: CTCAGAATTCCCAGCAGTGC R: ATTTGCATCCTGGTTCCGTG |
| *ACTRT3* | F: TTATGCACCTTGAGACCCCT R: GGCAGGCACCATCTTTGCTA |
| *KLF5* | F: CCTTGCACATACACCATGCC R: CGTTTCTCCAAATCGGGGTT |
| *SAXO1* | F: GCATCATTGTCCACACCTCC R: TTGTGAGGCCCAAAGTCTCT |
| *PLCZ1* | F: TTGACTGCTGGGATGGATCA R: CTGTCGGCCATCACTTCTTG |
| *TXNDC8* | F: TGTCTGTGCAATACCGAAGC R: GATGTGGTAAGTTTGGGCCA |
| *CHD5* | F: TGTTCAACATTGCAGACGGG R: AGACTTGAAGGGCTCGTTGA |
| *TNP1* | F: GGGGCAAGAACAGAACTCCT R: GCATCGTCACAGCGTTTTCT |
| *SPEM1* | F: CCTGAGGATTGGGAGGGTTT R: AGGGTACACGTAGGCCTCTA |
| *SPATA19* | F: CCAAAGTCAGCTCGGATGTG R: GCTCGGCACTTGGAAGATAC |
| *GAPDHS* | F: CCAGCCTACCCCATACTCAG R: CCACTCGGTTACTGTAGCCA |
| *AKAP1* | F: GGCTACAAGAGGGTGAAGGT R: CCACGCTCCACAGTTGAATC |
| *THEG* | F: GACCACTTCTATCTGGCCCA R: CCAGGTGAAGAAGGCGGTTA |
| *NR5A1* | F: GCTACCTCTACCCTGCCTTC R: ACTTGGTCCTCATCTGGCTC |
| *CYP11A1* | F: CACTTGTACGAGATGGCACG R: TGTCTTGGCAGGAATCAGGT |
| *STAR* | F: CGAGACTTTGTGAGCGTACG R: GGTTGATGATGGTCTTCGGC |
| *ACTA2* | F: AGGGAGTGATGGTGGGAATG R: CTCGGTGAGAAGGGTTGGAT |
| *PECAM1* | F: GACTCATTGCAGTGGTGGTC R: CCAAGGTCAGGGGCTCTTTA |
| *CX3CR1* | F: CATCACCGTTATCAGCGTCG R: TCTCAGGGTAGTCGCCAAAG |
| *CD68* | F: GCAGTGCAGTGGACATTCTC R: GAAACTTGGTCCAAAGGCCC |
| *CD163* | F: GGCAACGAGTCCCATCTTTC R: CCCCAGGTCCCAAGAATCTT |
| *TNF* | F: TTCCTCACCCACACCATCAG R: ATCCCAAAGTAGACCTGCCC |
| *IL10* | F: TTCTGCCCTGCGAAAACAAG R: TGTAGACACCCCTCTCTTGG |
