# Supplementary material for: Single-cell transcriptome analysis and in vitro differentiation of testicular cells reveal novel insights into male sterility of the interspecific hybrid cattle-yak
Source: BMC Genomics. 2023 Mar 27;24:149. doi: 10.1186/s12864-023-09251-2 (PMC10045231; doi:10.1186/s12864-023-09251-2)
Supplement: Supplementary file 5 — Additional file 5: Table S5. Primer sequences for detection of germ cell marker genes during spermatogenic cell proliferation and differentiation between cattle-yak and yak. [file 12864_2023_9251_MOESM5_ESM.docx]

**Table S5 Primer sequences for detection of germ cell mark genes during spermatogenic cell proliferation and differentiation between cattle-yak and yak**

| Gene name | Primer sequence（5′→3′） |
| --- | --- |
| *DAZL* | F: GCTTATCATGTGCAGCCACG R: ACTCCTTTGTTCCCCAGCAG |
| *BOLL* | F: ATTGCTATGCCTGCACCAGT R: GCTGGATCTCGGCTACACAA |
| *STRA8* | F: GCACATCTAGCCCCAGTACC R: CATCGTCGACAGGAAGGTCC |
| *SYCP3* | F: AATGGTGCCCTCTGGAAGAA R: AAGTGTTGGGGTCTTCTCTTCG |
| *UCHL1* | F: CTGGAGGAGGAGTCTCTGGG R: GAAGTTCTCATGCTGCGCC |
| *AKT1* | F: TTCTCCGTGGCCCAATGC R: GACCGGAAGTCCATCGTCTC |
| *THY1* | F: GCCCTCCTGCTAACAGTCTTAC R: GGCAGTCTAGACGAAGGCTC |
| *GFRA1* | F: CCTTGTGCAAACTTACAGGCAC R: GCTCCTCAGAGTCTGCTGTTG |
| *c-kit* | F: TCTCAGAGAGCACCAATCATATTT R: ATGCAGACTGCTTCAGACATC |
| *KI67* | F: TTTGGGGAAGCAGAGGGTCT R: GTTTCTCCTGTTGCTTGGTCG |
| *Acrosin* | F: AACAGCTATGTGGTCGTGGG R: TAGCTTGCTGAGAGGGAGGT |
| *mTOR* | F: AGCAACAACCCTCGCAAGAT R: CACTGTGATCTCCTCTGTGC |
