# Supplementary material for: Single-cell transcriptome analysis and in vitro differentiation of testicular cells reveal novel insights into male sterility of the interspecific hybrid cattle-yak
Source: BMC Genomics. 2023 Mar 27;24:149. doi: 10.1186/s12864-023-09251-2 (PMC10045231; doi:10.1186/s12864-023-09251-2)

**Supplementary Information for**

Single-cell transcriptome analysis and in vitro differentiation of testicular cells reveal novel insights into male sterility of the interspecific hybrid cattle-yak

TserangDonko Mipam^1^, Xuemei Chen^1^, Wangsheng Zhao^2^, Peng Zhang^1^, Zhixin Chai^1^, Binglin Yue^1^, Hui Luo^1,2^, Jikun Wang^1^, Haibo Wang^1^, Zhijuan Wu^1^, Jiabo Wang^1^, Mingxiu Wang^1^, Hui Wang^1^, Ming Zhang^1^, Hongying Wang^3^, Kemin Jing^1^, Jincheng Zhong^1*^, Xin Cai^1*^

*Corresponding author. Email: caixin2323@126.com (XC); zhongjincheng518@126.com (JCZ)

**This PDF file includes:**

Fig. S1 to S6

Original pictures and supplementary information for Western Blotting


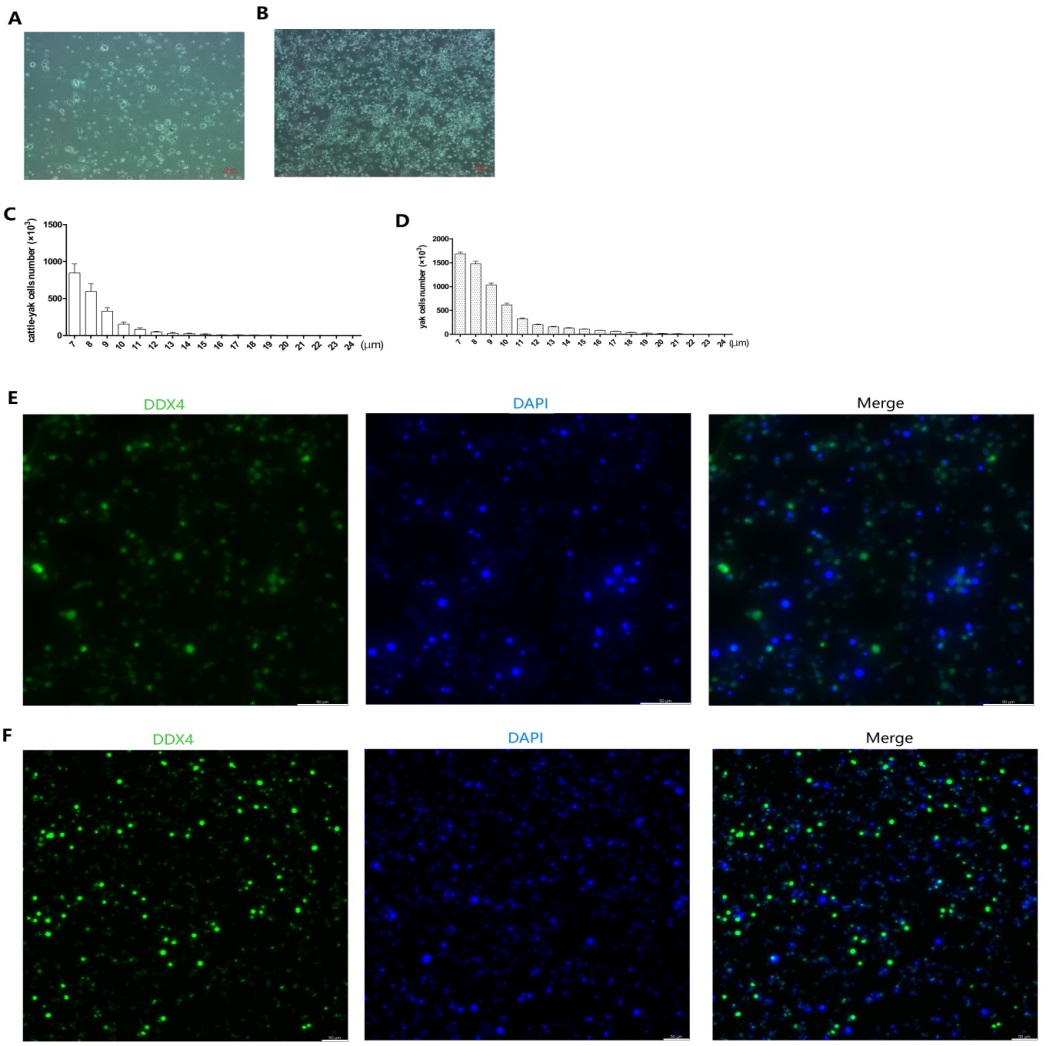


**Fig. S1 Identification of pubertal testicular cells from cattleyak and yak.** (A and B) The re-suspension cells of cattleyak and yak after 2 days of culture, respectively. (C and D) The size and distribution of testicular cells from cattleyak and yak, respectively. (E and F) Immunofluorescence of DDX4 (green) in pubertal testicular cells of cattleyak and yak, respectively. The scale bars represent 50 μm.


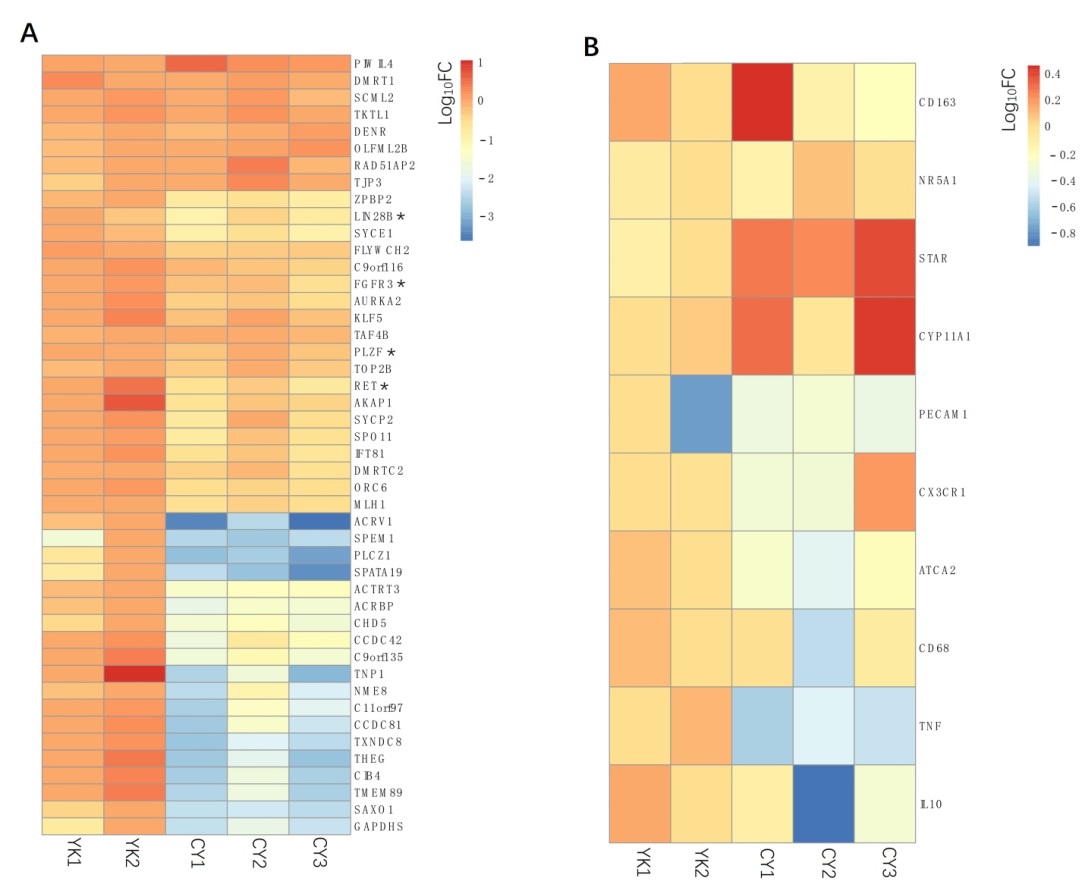


Fig. S2 Validation of cell type specific gene expressions between testicular cells of cattleyak and yak. (A) Log_10_ fold change values for RT-qPCR detection of 46 germ cell specific signature gene expression in pubertal testes between cattleyak and yak. (B) Log_10_ fold change values for RT-qPCR detection of 10 niche cell type specific signature gene expression in pubertal testes between cattleyak and yak. YK and CY denote yak and cattleyak, respectively. * indicates the differentially expressed signature genes in undifferentiated spermatogonial cells.


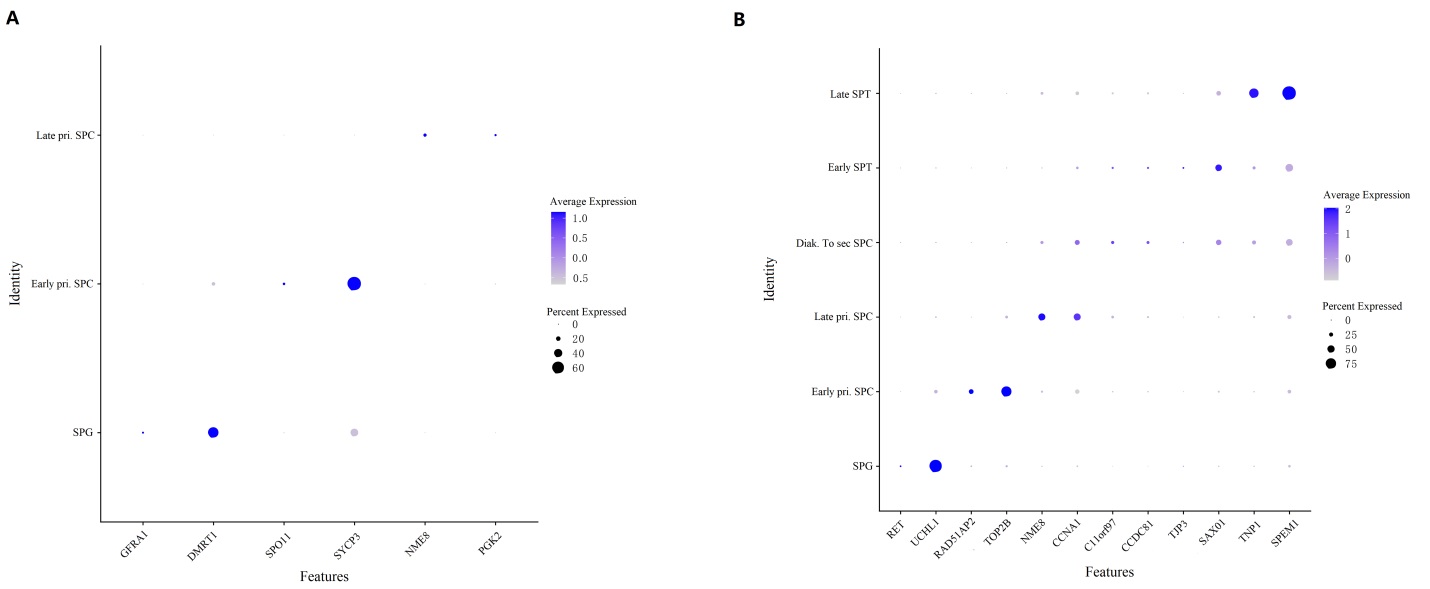


Fig. S3 Dot plot showing relative expression patterns of the potential marker genes for each germ cell type (A) Relative expression of the potential marker genes for spermatogenic cells in cattleyak. (B) Relative expression of the potential marker genes for spermatogenic cells in yak.


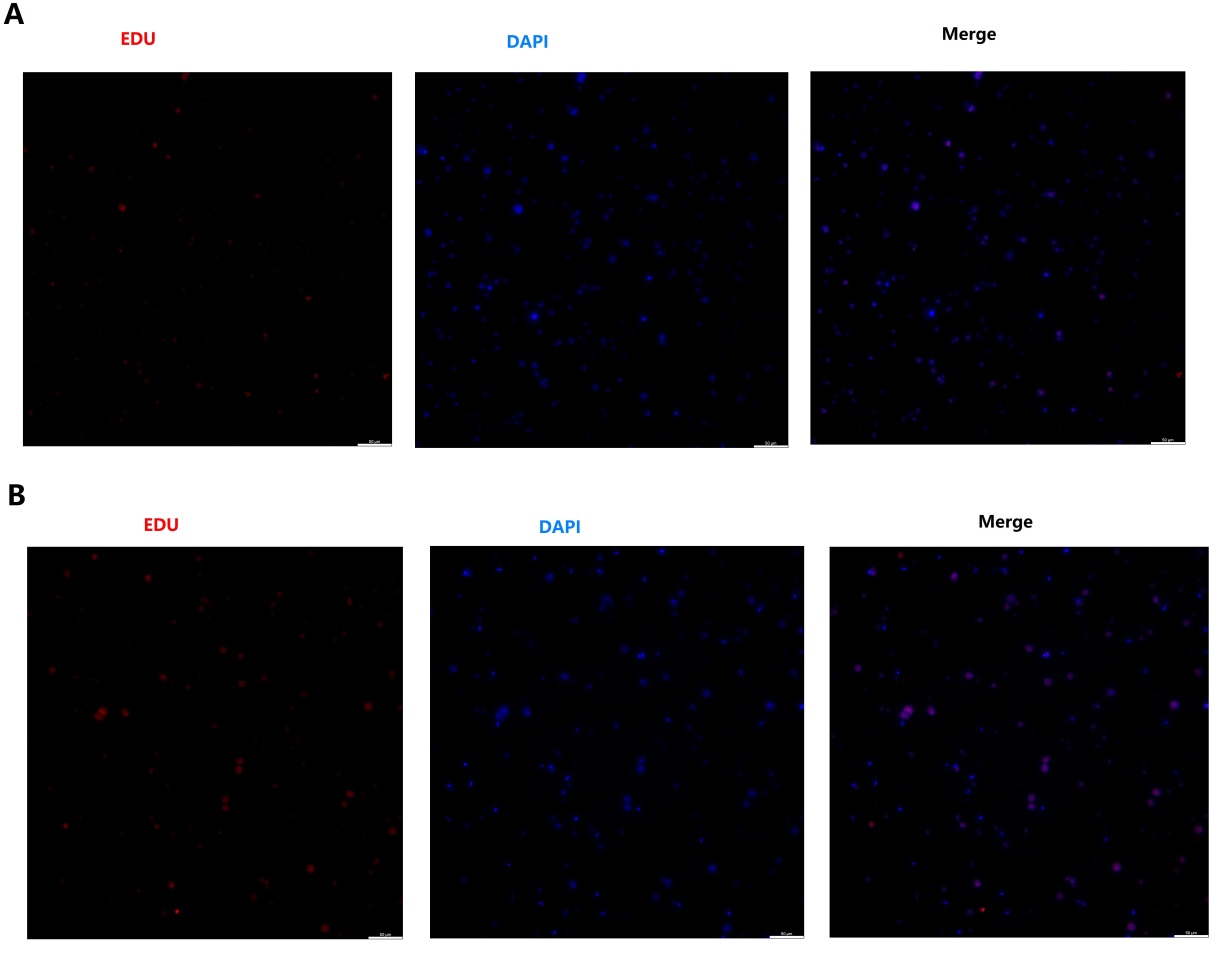


Fig. S4 Proliferation analysis of the spermatogenic cells cultured *in vitro* by EDU staining. (A, B) EDU and DAPI staining of cattle-yak and yak spermatogenic cells, respectively.


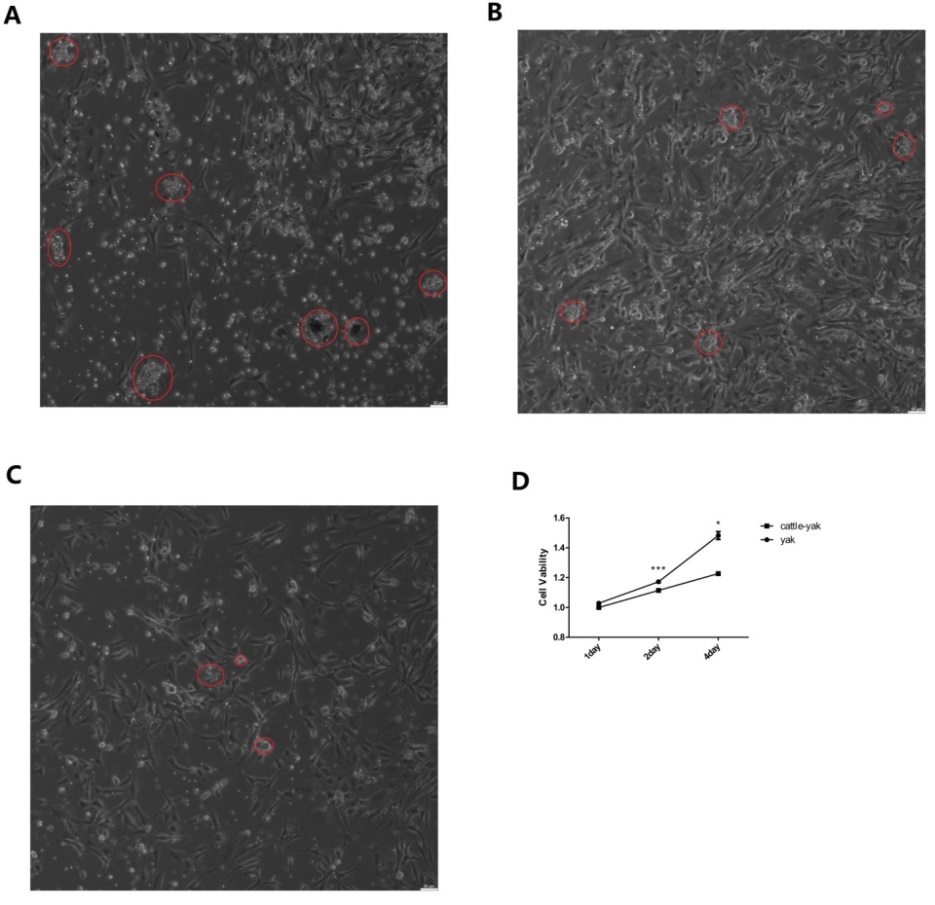


**Fig. S5 Spermatogenic cells co-cultured with different testis somatic cells and cell viability analysis.** (A) Yak spermatogenic cells co-cultured with yak testicular somatic cells as feeder and sub-cultured three times *in vitro*. (B) Cattleyak spermatogenic cells co-cultured with cattleyak testicular somatic cells as feeder and sub-cultured three times *in vitro*. (C) Cattleyak spermatogenic cells co-cultured with yak testicular somatic cells as feeder and sub-cultured three times *in vitro*. (D) CCK8 assay detected the viability of yak and cattleyak spermatogenic cells co-cultured with their testicular somatic cells for 4 consecutive days. The scale bars represent 50 μm. * denotes P<0.05, and *** denotes P<0.001.


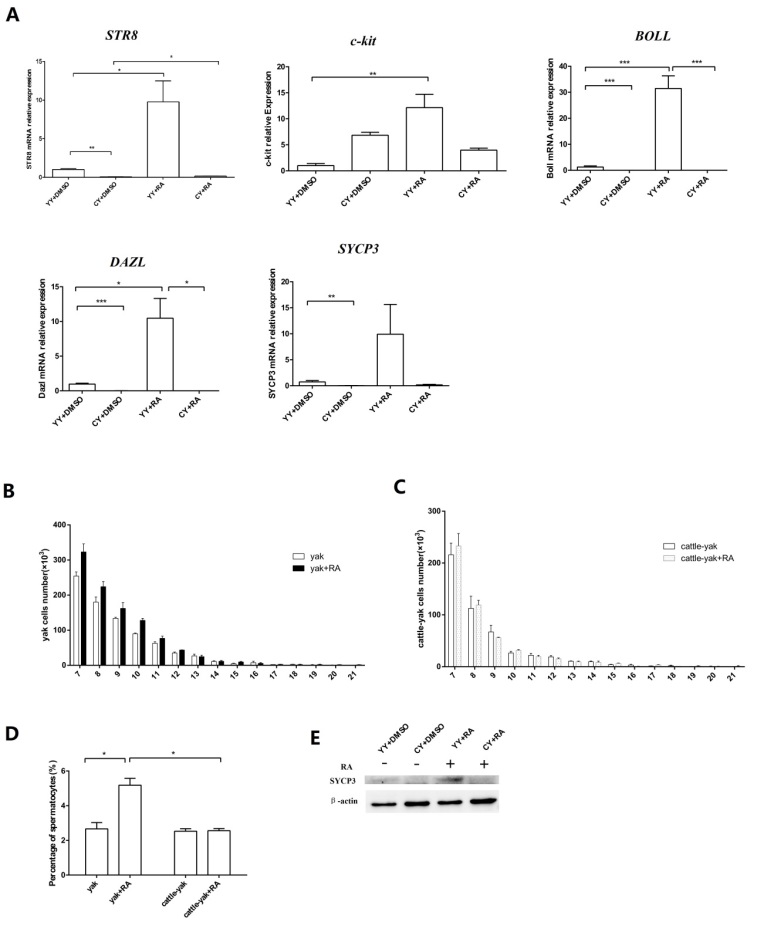


**Fig. S6 Inducion of** **spermatogenic cells of cattleyak and yak with retinoic acid (RA) *in vitro****.* (A) *STR8, c-kit, BOLL, DAZL* and *SCYP3* mRNA expressions in of spermatogenic cells of cattleyak and yak after AR inducion. (B and C) The cell size and distribution of spermatogenic cells after AR induction in yak and cattle yak, respectively. (D) Percentage of spermatocytes of spermatogenic cells after AR inducion in yak and cattle yak, respectively. (E) SYCP3 protein expression in spermatogenic cells after AR induction in yak and cattleyak, respectively.

YY and CY denote yak and cattleyak, respectively. ***** denotes P<0.05, ** denotes P<0.01 and *** denotes P<0.001. The expression of β-actin was served as control.

**Original pictures of western blotting:**

1. **PLZF (Fig. 4E)**

The antibody information: Santacruz, sc-28319, 80-90 KD

Original picture
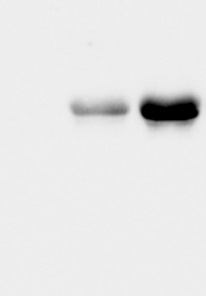


Supplementary information (Merge of pre-stained protein ladder with chemiluminescence bands)


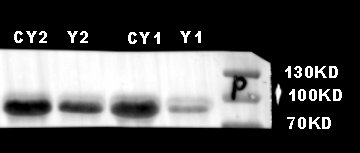


**2）GFRA1 (Fig. 4E)**

The antibody information: Abcam, ab84106, 55 KD

Original picture:


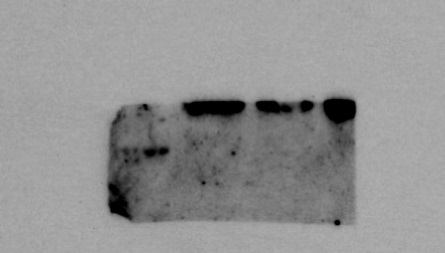


Supplementary information:


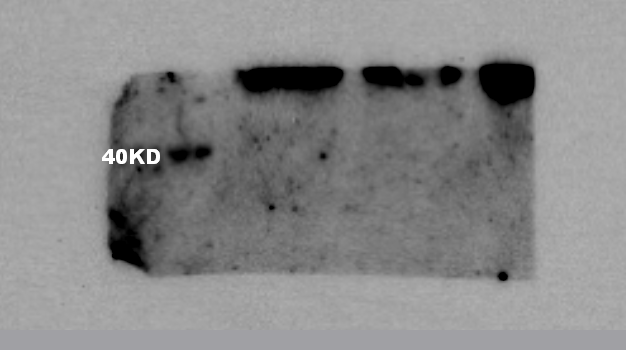


**3）c-kit (Fig. 4E)**

The antibody information: Abcam, ab111033, ~120 KD

Original picture:


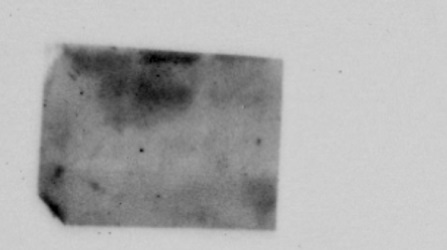


Supplementary information:


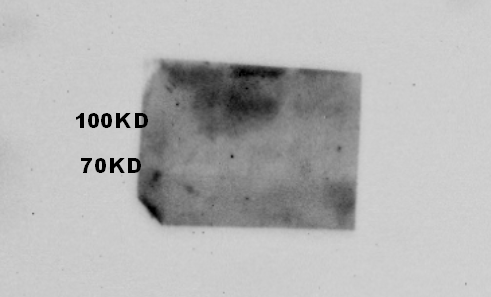

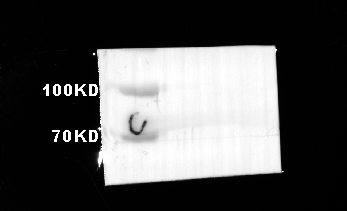


**4）DDX4 (Fig. 4E)**

The antibody information: Abcam, ab13480,76 KD

Original picture:


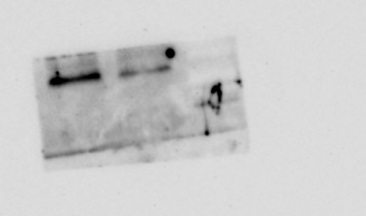


Supplementary information:


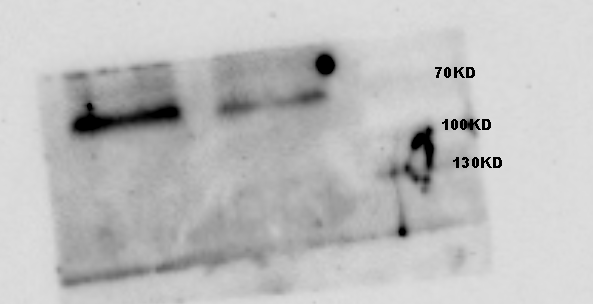


**5）β-actin (Fig. 4E)**

The antibody information: Bioss, bs-0061R, 42 KD

Original picture:


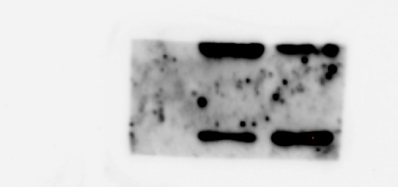


Supplementary information:


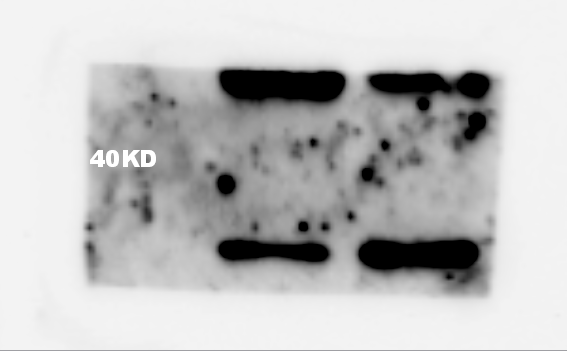


**6）SYCP3 (Fig. S5E)**

The antibody information: Novus, NB300-232SS, ~28 KD

Original picture:


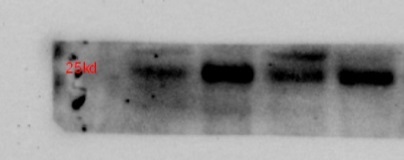


Supplementary information:


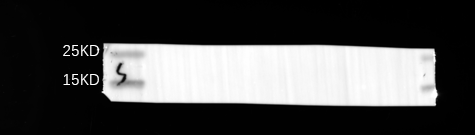

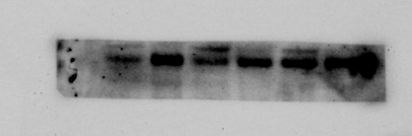


(Merge of pre-stained protein ladder with chemiluminescence bands)


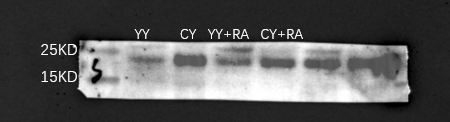


**7）β-actin (Fig. 5SE)**

The antibody information: Bioss, bs-0061R, 42 KD

Original picture:


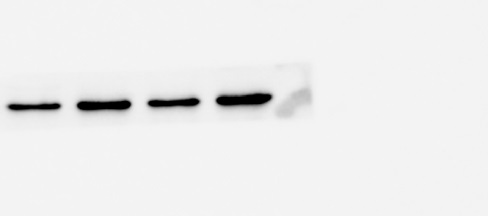


Supplementary information:


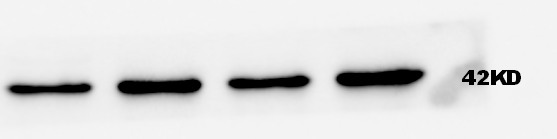

Supplement: Supplementary file 6 — Additional file 6: Fig. S1. Identification of pubertal testicular cells from cattleyak and yak. (A and B) The re-suspension cells of cattleyak and yak after 2 days of culture, respectively. (C and D) The size and distribution of testicular cells from cattleyak and yak, respectively. (E and F) Immunofluorescence of DDX4 (green) in pubertal testicular cells of cattleyak and yak, respectively. The scale bars represent 50 μm. Fig. S2. Validation of cell type specific gene expressions between testicular cells of cattleyak and yak. (A) Log10 fold change values for RT-qPCR detection of 46 germ cell specific signature gene expression in pubertal testes between cattleyak and yak. (B) Log10 fold change values for RT-qPCR detection of 10 niche cell type specific signature gene expression in pubertal testes between cattleyak and yak. YK and CY denote yak and cattleyak, respectively. * indicates the differentially expressed signature genes in undifferentiated spermatogonial cells. Fig. S3. Dot plot showing relative expression patterns of the potential marker genes for each germ cell type (A) Relative expression of the potential marker genes for spermatogenic cells in cattleyak. (B) Relative expression of the potential marker genes for spermatogenic cells in yak. Fig. S4. Proliferation analysis of the spermatogenic cells cultured in vitro by EDU staining. (A, B) EDU and DAPI staining of cattle-yak and yak spermatogenic cells, respectively. Fig. S5. Spermatogenic cells co-cultured with different testis somatic cells and cell viability analysis. (A) Yak spermatogenic cells co-cultured with yak testicular somatic cells as feeder and sub-cultured three times in vitro. (B) Cattleyak spermatogenic cells co-cultured with cattleyak testicular somatic cells as feeder and sub-cultured three times in vitro. (C) Cattleyak spermatogenic cells co-cultured with yak testicular somatic cells as feeder and sub-cultured three times in vitro. (D) CCK8 assay detected the viability of yak and cattleya [file 12864_2023_9251_MOESM6_ESM.docx]
